# Fabrication of cerium titanate cellulose fiber nanocomposite materials for the removal of methyl orange and methylene blue from polluted water by photocatalytic degradation

**DOI:** 10.1007/s11356-022-21430-4

**Published:** 2022-06-23

**Authors:** Yousra H. Kotp

**Affiliations:** Water Treatment & Desalination Unit, Hydrogeochemistry Department, Desert Research Center, El-Matariya, Cairo, B 11753 Egypt

**Keywords:** Cerium titanate (Ce-Ti), Cellulose fiber (Cf), Photocatalytic degradation, Sunflower seed husk, Dyes

## Abstract

In this study, cellulose fibers (Cf), extracted from sunflower seed husk, and different molar ratios of cerium titanate (Ce-Ti) NPs were prepared from sunflower seed husk extract by a green biosynthesis approach. Cf and Ce-Ti NPs were reacted via cross-linking reaction to synthesize a novel nanocomposite photocatalyst of Ce-Ti/Cf. Using Fourier-transform infrared (FTIR), X-ray diffraction (XRD), and scanning electron microscopy (SEM-EDX) spectroscopy, all manufactured materials were characterized. The results obtained from FTIR and EDX analyses indicated that Cf and its nanocomposites (0.1 Ce-Ti/Cf, 0.3 Ce-Ti/Cf, and 0.5 Ce-Ti/Cf) were successfully prepared by harnessing biomass extract from sunflower seed husk. Furthermore, XRD revealed that the degree of crystallinity of the nanocomposites was enhanced by increasing the molar ratios of the Ce-Ti NPs. The photocatalytic activity of as-fabricated 0.1 Ce-Ti/Cf, 0.3 Ce-Ti/Cf, and 0.5 Ce-Ti/Cf nanocomposite samples was investigated on methylene blue (MB) and methyl orange (MO) dyes as model organic compounds found in wastewaters. The effects of dose, contact time, and pH on the photocatalytic activity of the synthesized nanocomposites, the photodegradation kinetic parameters of MB, and MO degradation with/without the addition of H_2_O_2_ were also studied. The results revealed that high photodegradation efficiency could be obtained as the ratio of TiO_2_ in the Ce-Ti nanocomposite formula increases. Moreover, after sunlight irradiation, the adsorption capacity and the dye decomposition ratio significantly increase during the early contact time and reach equilibrium at about 240 and 120 min for 0.5 Ce-Ti/Cf nanocomposite photocatalyst in the absence and presence of hydrogen peroxide, respectively. In light of the obtained results and the practical wastewater treatment study conducted, the prepared photocatalyst from Ce-Ti/Cf nanocomposites could be a promising material for treating dye wastewater especially collected from Egypt.

## Introduction

Nowadays, wastewater is a serious issue that faces humankind because of technological development and population growth. The release of various wastes into the environment, such as herbicides, phenolic compounds, synthetic dyes, and detergents, has a significant impact on human life and the environment (Pirhashemi and Habibi-Yangjeh 2017). Chemical and biological treatments can be utilized to remove certain organic compounds, but their degradation by-products are not degradable and may be harmful (Aksu 2005; Yan et al. 2011; Zhang et al. 2014; Peng et al. 2016). The major organic chemicals classified according to their molecular structure are dyes, which are widely utilized in many industries, like leather, cosmetics, plastics, textiles, rubber, paper printing, leather, paints, and plastics. Because of their complex structure, dyes are usually challenging to degrade. They can cause many disorders in animals and humans, such as irritation, allergies, dermatitis, and even cancer (Zhang et al. 2014; Peng et al. 2016; Capanema et al. 2018). Therefore, the reusing of discharged wastewater has become the main target to save the environment over the world (Pirhashemi and Habibi-Yangjeh 2017).

To minimize water pollution, various chemical and physical treatment techniques can be utilized to remove dyes. These technologies involve Fenton chemical oxidation (Woo et al. 2014), electrochemical degradation (Fan et al. 2008), cation exchange membranes (Wu et al. 2008), bioremediation (Khataee et al. 2012), and photocatalysis (Jawad et al. 2015; Jawad et al. 2016). However, the performance of these methods is different, and there are disadvantages such as high cost, abundant toxic by-products (Cheng et al. 2020), and high energy consumption. Therefore, the photocatalytic method has the advantages of low energy consumption, environmental friendliness, and no selectivity in the degradation of different pollutants, and has attracted the attention of most researchers (Dong et al. 2015; Fagan et al. 2016a).

However, semiconductor photocatalysts have recently attracted great interest because of their possible application to detoxify environmental pollutants (Tryk et al. 2000; Alvaro et al. 2010). In particular, the importance of using semiconductor photocatalysts to degrade organic pollutants has stimulated tremendous efforts in its synthesis and characterization methods, making it an integral part of photocatalysis research (Mills and Hunte 1997; Herrmann 1999; Pera-Titus et al. 2004; Mohamed et al. 2012). Among photocatalysts, titanium dioxide (TiO_2_) has become a widely used semiconductor material, with low price, non-toxicity, and long-term stability because of its excellent photoreactivity. The photocatalytic activity of TiO_2_ depends on several factors, like surface area, crystallinity, impurities, and density of surface hydroxyl groups. TiO_2_ can be utilized as a photocatalyst for rutile crystal structures and anatase. The activity of the anatase phase is much higher than that of rutile (Lv et al. 2011), but it needs ultraviolet radiation to be activated photocatalytically. At present, the photo catalysis of titanium dioxide under visible light has aroused people’s interest in the use of sunlight by harnessing it (Barndõk et al. 2013).

The modification of TiO_2_ with metals has made it possible to synthesize visible light active photocatalysts. However, some dopants increase considerably the photocatalytic degradation of contaminants while others reduce it since they can act as recombination sites that lead to a reduction of quantum efficiency (Pelaez et al. 2012). For example, Byrne et al have demonstrated the high efficiency of W-doped TiO_2_ (Byrne et al. 2021) and the poor catalytic activity of Cu-doped TiO_2_ which arises from the charge recombination at defect sites that results from the incorporation of copper into TiO_2_ (Byrne et al. 2019a). Non-metal doping is an effective method for the preparation of high-temperature stable anatase TiO_2_ photocatalysts as well as co-doping. Fagan et al. (2016b) used an efficient, rapid, and straightforward method for the preparation of nitrogen and fluorine (N, F) co-doped high-temperature stable anatase using a microwave pre-treatment. Also, Fagan et al. (2016c) improved the high-temperature stability of anatase TiO_2_ photocatalysts by N, F, and P co-doping. For example, it is known that the modification of anatase TiO_2_ with hexagonal boron nitride increases the anatase to a rutile transition temperature and enhances photocatalytic activity under solar conditions in comparison to bare TiO_2_ (Byrne et al. 2019b). Although a large number of mesoporous TiO_2_ powder and film applications have been reported thus far, there are still two major deficiencies that hinder catalyst efficiency: (i) its broadband energy only allows activation in the ultraviolet region (about 3–5% of the total solar spectrum) and (ii) the rapid recombination of photogenerated charges (Barndõk et al. 2013; Zhang et al. 2017). One of the effective methods to eliminate the second deficiency in TiO_2_ photocatalytic activity is to modify TiO_2_ with various elements, especially rare earth metal ions and transition metals (Zhang et al. 2017). Thus, several previous studies have taken a multi-approach to prepare cerium titanate (Ce-Ti) nanoparticles, nanorods, porous, and cerium-doped TiO_2_ (Otsuka-Yao-Matsuo et al. 2004; Pei et al. 2015; Zhang et al. 2018; Vieira et al. 2018; Wang et al. 2019). The band gap state produced by Ce, which is used as a doping component in TiO_2_, improves the photocatalytic efficiency of such a type of catalyst (Barrio et al. 2012). So, a further research on this topic is needed. To the author’s best knowledge, no previous study mentioned the use of natural sunflower seed husk extract as a reducing agent to make photocatalytic cerium titanate nanoparticles.

However, the use of nanoparticles as catalysts generally has disadvantages, such as stirring to avoid precipitation, will be poisoned, and being lost in water when it is employed in flowing wastewater, resulting in difficult recovery as well as secondary pollution. To overcome these drawbacks, there are immobilization techniques, such as chemical vapor deposition (Zhang and Griffin 1995), sputtering (Sproul et al. 1997), polymer immobilization (Malynych et al. 2002), sol-gel (Brinker and Scherer 1990; Hu et al. 2008), and synthesis inside porous silica (Zhu et al. 2009). In the current work, biodegradable cellulose is selected as mechanical support to fix the nanoparticles. Cellulose is generally a natural biodegradable polymer on earth, renewable, cheap, and yearly cellulose produced from plants of about (1012 tons) and widely utilized to make fibers and gels (Capanema et al. 2018). The current biodegradable forms based on cellulose reduce their practical applications. Compared with them, biodegradable fiber materials show irreplaceable advantages in removal rate and adsorption capacity owing to their perfect solid-liquid separation performance, superior wastewater treatment performance, and fast water filtration speed (Shao et al. 2021). Single fiber production is cheaper than nano-scale fibrils and has admirable features such as improved capacity, good dispersion behavior, biodegradability, higher crystallinity, good thermal stability, and high performance. Nowadays, many studies on the extraction of individual fibers from different lignocellulosic biomass sources seem to explore their applications in many effective applications (Sanchez-Garcia et al. 2008; Reddy et al. 2014; Puttaswamy et al. 2017).

However, this interest also includes finding new sources of biomass to produce cellulosic fibers for large-scale applications. Therefore, sunflower husk can be a very attractive source of residual agricultural biomass for the production of individual fibers. Sunflower (*Helianthus annuus*) is an essential oil seed crop globally, and its production is second only to peanuts and soybeans (Byrareddy et al. 2008). The current consumption of sunflower seeds or their by-products as human food is very low and underutilized. However, its usage as animal feed is very extensive and continues to grow. Sunflower seeds are mainly utilized as snacks for humans, roasting the seeds into peanuts and chestnuts for consumption. Peeled sunflower seeds are widely included in vegetarian diets and are mainly sold in health food stores as an effective protein alternative source (Nwokolo 1996). Sunflower seeds are covered with a very fibrous shell that makes up about 15–25% of the seeds. These hooves are mainly used as litter for cattle. Because of its needle-like nature, a small amount of sunflower husk can be added to animal feed as a source of fiber, which can damage the gastrointestinal tract. This experiment uses sunflower hulls as a supplementary fuel for coal-fired power plants (Crum et al. 1992). A preliminary study on natural sunflower hull fibers was reported in the literature, and it was found that these fibers contained about 25.7% cellulose and reducing sugars (Taha et al. 2012). To our knowledge, there are no reports on the extraction of Cf from natural sunflower husks. Therefore, functionalizing cellulose fibers with Ce-Ti hybrid to obtain a renewable and highly efficient nanocomposite to decompose dye pollutants under sunlight irradiation is worth investigating.

In this work, a highly efficient sunlight-responsive photocatalyst was prepared using cellulose (Cf) fibers, CeO_2_-NPs, and various ratios of Ce-Ti nanoparticles that had previously been prepared using green synthesis techniques. Next, the Ce-Ti/Cf nanocomposite was prepared with a highly efficient degradation capacity of MB and MO dyes. In this regard, Cf and Ce-Ti NPs were prepared from sunflower seed husk and its ethanolic extract, respectively, through a green biosynthesis approach. Then, the cellulose fiber (Cf) nanocomposite photocatalyst was successfully fabricated using a cross-linking reaction between cellulose fiber (Cf) and a different molar ratio of cerium titanate (Ce-Ti) nanoparticles to obtain 0.1 Ce-Ti/Cf, 0.3 Ce-Ti/Cf, and 0.5 Ce-Ti/Cf nanocomposites photocatalysts. Finally, the prepared Cf, CeO_2_-NPs, 0.1 Ce-Ti/Cf, 0.3 Ce-Ti/Cf, and 0.5 Ce-Ti/Cf nanocomposite photocatalysts were characterized by FTIR, XRD, SEM-EDX, and elemental analysis measurements. Also, the photocatalytic activity of the 0.1 Ce-Ti/Cf, 0.3 Ce-Ti/Cf, and 0.5 Ce-Ti/Cf nanocomposite materials were evaluated under sunlight irradiation using MO and MB as models. Possible pathways and mechanisms for the photodegradation of the prepared photocatalysts were discussed. Eventually, the improved nanocomposite fibers were used in the photodegradation processes of polluted real water collected from Egypt.

## Experimental

### Materials

Cellulose fibers were extracted from sunflower seed husk waste, directly collected from a local market in Egypt. All chemicals that utilized in this work were purchased from Sigma-Aldrich Co. (e.g., titanium (IV) isopropoxide (M.Wt = 288.25 g mol^−1^, purity ≥ 97.0%), ammonium cerium nitrate (M.Wt = 548.26g mol^−1^, purity ≥ 98.0%), sodium hydroxide solid (M.Wt = 39.99g mol^−1^ , purity ≥ 98.0%), sodium hypochlorite (M.Wt = 74.44g mol^−1^, purity ≥ 98.0%), ammonium persulfate (M.Wt = 228.18g mol^−1^, purity ≥ 98.0%), glutaraldehyde (M.Wt = 100.11g mol^−1^, purity ≥ 99.0%), ethanol (purity ≥ 99.0%), acetic acid (purity ≥ 98.0%), methylene blue dye (MWt = 319.85, purity ≥ 98.0%), and methyl orange (MWt = 327.33, purity ≥ 98.0%), as the model dye for photocatalytic degradation.

### Preparation of cerium oxide and cerium titanate NPs using sunflower seed husk extract

#### Preparation of sunflower seed husk extract

The husks of sunflower seeds were washed thoroughly with distilled water for removing adhering soil. Before the extraction process, the washed shells were dried in the sun to evaporate water for 72 h; the dried sunflower seed husk was ground and sieved. Then, about 50 g of ground powder was treated with 400 mL ethanol. The mix was retained at 25 °C for 24 h with continuous shaking and filtered using refinery paper. Then, wash the sunflower husks with distilled water and dry them in an oven at 80 °C for 24 h.

#### Preparation of cerium oxide NPs

For preparing CeO_2_ NPs, 100 mL of sunflower seed husk extract was heated at 80 °C for 1 h, and then, 0.01 M of ammonium cerium nitrate aqueous solution was added. Cerium hydroxide was precipitated by adding little drops of ammonium hydroxide (NH_4_OH) solution until the pH was maintained at 8.6 and reduced to a dark orange solution. The precipitate was filtered and annealed at 500 °C for 3 h. A yellow precipitate was formed and packed carefully for further characterization.

#### Preparation of cerium titanate NPs with different molar ratios of titanium

Typically, 0.01 M of ammonium cerium nitrate and a different molar ratio of titanium (IV) isopropoxide (0.1, 0.3, and 0.5 M) were dissolved in 100, 200, and 300 mL of sunflower seed husk extract, respectively. The solution was refluxed for 1 h with vigorous stirring at 80 °C. Cerium titanate was precipitated by adding little drops of ammonium hydroxide (NH_4_OH) solution until the pH was maintained at 7.6, 8.6, and 9.5 for 0.1, 0.3, and 0.5 M, respectively. The precipitate was filtered, washed with DI water, and annealed at 500 °C for 3 h. A light-yellow precipitate was obtained and packed carefully for further characterization.

### Preparation of microcrystalline cellulose fiber from the treated sunflower seeds husk

**Scheme 1:**
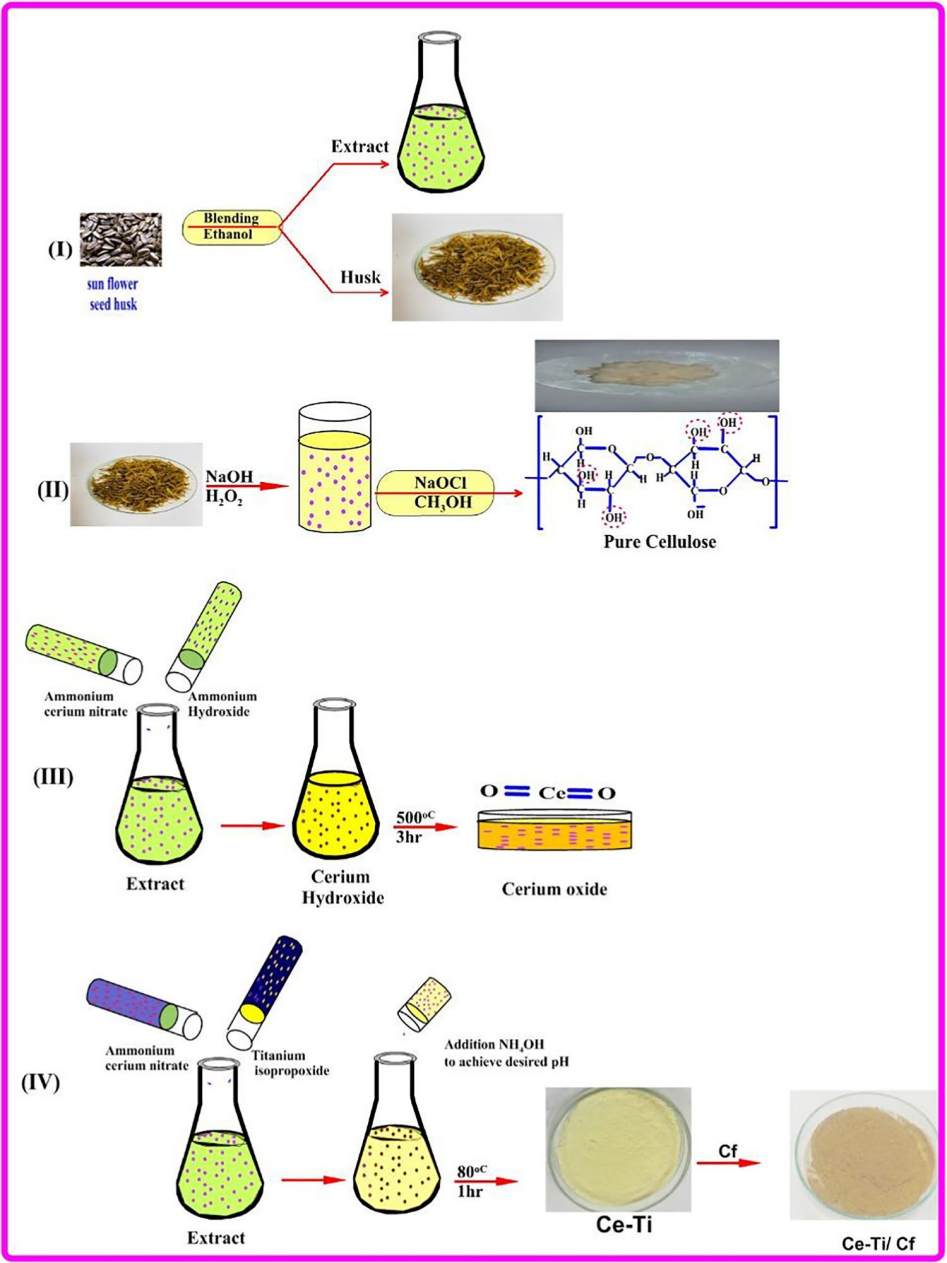
The schematic diagram for the preparation procedures of Cf, CeO_2_ NPs, Ce-Ti NPs, and Ce-Ti/Cf nanocomposite materials

The sunflower seed hulls pre-treated with 100 g ethanol were washed many times with distilled water and then dried in an oven at 80 °C for 24 h. The pre-treated waste material was further digested with 18% (w/w) NaOH alkaline solution at 110 °C for 1 h. After digestion, the filtered pulp is thoroughly washed until there is no alkali residue, and the pH reaches 7 and then dried at 80 °C. The bleaching method was performed by the method characterized by Alabi et al. (2020) but with a slight modification. In the bleaching procedure, 30 g of the air-dried samples was placed in a 5-L Erlenmeyer flask, and 1000 mL of 700 chlorites and 300 mL of 90%v/v acetic acid were added. The mix was heated in a 70 °C water bath for 1 h with continuous stirring. After processing for 1 h, drain the sample, add 750 mL of hydrogen peroxide, and heat it in water at 70 ° C for 1 h with continuous stirring. After treatment, the sample was drained and then extracted with 600 mL of 5% w/v NaOH at 70 °C for 1 h. After the sample was extracted with distilled water, the alkali was washed off. This process was carried out three times in sequence and dried in an oven at 80 °C to obtain pure cellulose fiber (Cf). Finally, the preparation scheme is presented in Scheme 1.

### Preparation of cerium titanate cellulose fiber (Ce-Ti/Cf) nanocomposite materials

First, cellulose fibers (Cf) were activated using phosphoric acid as follows: 5 g of cellulose fibers was mixed with 200 mL of distilled water, and then, 2 mL of 2M phosphoric acid was added and stirred with vigorous stirring. The mix was retained at 25 °C for 6 h; Cf was filtered, washed with DI water, and dried at 50 °C for 24 h. Second, activated Cf was intercalated using different molar ratios of cerium titanate, as follows: 1 g of the activated Cf sample was dispersed in 100 mL of distilled water. Then, 0.08 g ammonium persulfate was put onto the previous suspension and heated at 80 °C for 2 h. Then, 0.2 g of (0.1, 0.3, and 0.5 M) cerium titanate previously prepared was added and continued for another 2 h at 80 °C. After mixing the components, the nanocomposite fiber was cross-linked by adding glutaraldehyde solution, and the pH of the solution was adjusted by using ammonium hydroxide solution and became 11.4, 11.7, and 12 for 0.1, 0.3, and 0.5 M cerium titanate, respectively. The cross-linked fiber was heated at 80 °C for 24 h, washed away with purified water, and dried at 70 °C. The tests were denoted by acronyms describing the composition of the Cf and the intercalated different molar ratio of cerium titanate; for example, 0.1 Ce-Ti/Cf, 0.3 Ce-Ti /Cf, and 0.5 Ce-Ti /Cf represent the Cf intercalated with 0.1 M Ce-Ti, 0.3 M Ce-Ti, and 0.5 M Ce-Ti, respectively.

### Characterization

The chemical functional groups exist in Cf, CeO_2_NPs, CeO_2_/Cf, and Ce-Ti loaded cellulose fiber-containing different molar ratios of titanium (0.1 Ce-Ti/Cf, 0.3 Ce-Ti/Cf, and 0.5 Ce-Ti/Cf) nanocomposite photocatalyst were analyzed with Fourier-transform infrared spectroscopy (FTIR). An FTIR spectrometer (Nicolet Avatar 230 spectrometer) was used to verify infrared spectra with wavenumbers between 400 and 4000 cm^−1^ at a rate of 30 scans per minute.

We use the wide-angle X-ray diffraction spectrum of Cu Ka radiation (*λ* = 0.1542 nm) (PAN Analytical X’pert Superior Score Plus diffractometer) (model: PW3040/60) to obtain the crystal size of Ce-Ti cross-linked Cf and exponential influence cellulose fiber crystallinity. The spectrum range is 10° to 70° in a step size of 0.05° (2θ) at 40 kV and 30 mA. Scherrer’s formula aids in measuring the cellulose crystals’ size in the fiber structure (Elenga et al. 2009), and the crystallinity index (CRI) was calculated from the height of the peak (Segal et al. 1959). Energy-dispersive X-ray spectroscopic analysis (EDX) spectroscopy was measured on an SEM instrument (an SEM Model Quanta field emission gun (FEG) with applying electrical power of 30 kV) (JEOL Company, Tokyo, Japan) equipped with an Oxford EDAX system. The elemental composition (C, H, N, and S) of Cf, CeO_2_ NPs, CeO_2_/Cf, 0.1 Ce-Ti/Cf, 0.3 Ce-Ti /Cf, and 0.5 Ce-Ti/Cf were analyzed by a Thermo Scientific Flash EA 1112. Samples were dried before measurements, and 2.5 mg of each sample was weighed in tin capsules, and the measurements were performed in duplicate.

### Sunlight photocatalytic activity test

Photocatalytic tests were performed in a batch experiment. All photocatalytic tests were conducted under the same conditions on sunny days between 8:30 AM and 3:00 PM (the reaction time was 6 h) from March to April. The ambient temperature was between 26 and 31 °C, and the dye solutions were prepared using deionized water. The same mass (150 mg) of 0.1 Ce-Ti/Cf, 0.3 Ce-Ti/Cf, and 0.5 Ce-Ti/Cf nanocomposite was placed into MO and MB aqueous solutions in a quartz tube and stirred for 60 min in the dark to reach the adsorption-desorption equilibrium. Then, the quartz tube was subjected to sunlight. Batch experiments were performed by varying many experimental variables like adsorbent dosage (0.01 to 0.2 g), pH (2.5 to 10), photodegradation time (0 to 300 min), and H_2_O_2_ addition to determining the better photocatalytic degradation states. The pH of MO and MB dyes was set by inserting either 0.10 mol/L NaOH or HCl. After adding, 0.1 Ce-Ti/Cf, 0.3 Ce-Ti/Cf, and 0.5 Ce-Ti /Cf nanocomposites, the solution’s concentration of MO and MB was monitored by an ultraviolet spectrophotometer (Elico EI 301E, India) at 465 nm and 664 nm, respectively. The % degradation of methyl orange or methylene blue dyes at time *t* was estimated through Eq. (1).

1 $$\mathrm{Degradation}\ \mathrm{rate}={C}_0-{C}_t/{C}_0\times 100$$

where *C*_0_ is the initial dye concentration (mg L^−1^) and C_t_ is the dye concentration (mg L^−1^) after time *t* (min).

### Wastewater treatment processes by photocatalytic degradation

The photocatalytic degradation test was carried out on the actual factory wastewater collected from the 10th of Ramadan city, Egypt, with a pH of 7.8 using batch technology under sunlight. Therefore, 1.50 g of each 0.1 Ce-Ti/Cf, 0.3 Ce-Ti/Cf, and 0.5 Ce-Ti/CF nanocomposites were added to 100 mL of actual wastewater and exposed to sunlight at an ambient temperature of 31 °C for 2 h under magnetic agitation. EC and pH values were assessed as described previously (Kotp 2021). Turbidity measurement techniques were used to measure sulfate (SO_4_^2−^) ions. Heavy metal ions were considered by ICPS (ICAP 6500 Duo, Thermo Scientific, England). The concentration of MO, MB, Congo red, Malachite green, and safranin in the solution was determined by an ultraviolet spectrophotometer (Elico EI 301E, India) at 465 nm, 664 nm, 497 nm, 624, and 473 nm, respectively. The mineralization efficiencies of samples by highly active samples at time *t* may be presented as (Capanema et al. 2018):

2 $$\mathrm{Mineralization}\ \mathrm{efficiency}=100\times \left(\mathrm{CO}{\mathrm{D}}_0-\mathrm{CO}{\mathrm{D}}_t/\mathrm{CO}{\mathrm{D}}_0\right)$$

where COD_0_ and COD_t_ are the initial and at time *t* COD values of the sample solutions.

### Statistical analysis

Analysis of variance (ANOVA) was utilized to assess the major essential effects and the interaction effects of parameters affecting the efficiency of the photodegradation process. For statistical significance, this work aimed to obtain the absolute rate of *t*-ratio to be > 2 or the *P*-value to be < the significance level (*α* = 0.05). The *p*-value was utilized to confirm the significance of every coefficient; if the p-value < 0.05, the model term will be significant.

## Results and discussion

### Characterization of Cf, CeO_2_ NPs, CeO_2_/Cf, and Ce-Ti loaded cellulose fiber-containing different molar ratios of titanium (0.1 Ce-Ti/Cf, 0.3 Ce-Ti/Cf, and 0.5 Ce-Ti/Cf) nanocomposite materials

**Fig. 1:**
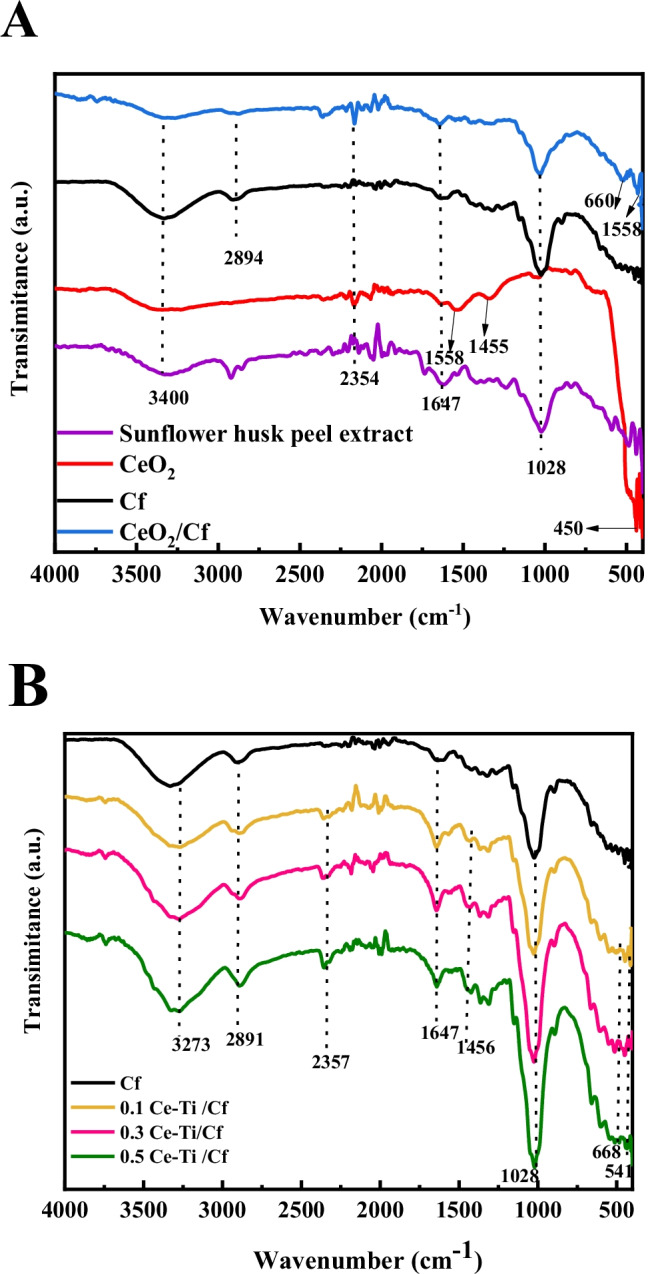
FTIR pattern for Sunflower seed husk extract, CeO_2_NPs, Cf, and CeO_2_/Cf (**A**) and (0.1 Ce-Ti/Cf, 0.3 Ce-Ti/Cf, and 0.5 Ce-Ti/Cf) nanocomposite materials (**B**)

FTIR analysis assessed the functional groups responsible for capping, reduction, and nanocomposite formation. Figure 1a shows the FTIR spectra of sunflower seed husk extracts, Cf, CeO_2_ NPs, and CeO_2_/Cf nanocomposite in the range of 400–4000 cm^−1^. As presented in the figure, the FTIR spectrum of the sunflower husk extract shows a clear peak at the wavelength of 3333 cm^−1^. The peak at 1610 cm^−1^ was related to the bond C = O stretching vibration of biomolecules involved in filtering (Fahma et al. 2010; Shawky et al. 2020), while the peak at 3333 cm^−1^ was related to the O-H stretching vibration of phenolic compounds. The band at 2922.5 cm^−1^ owed to the stretching vibration of the CH of all hydrocarbon components in polysaccharides (Fahma et al. 2010). The FTIR spectra of CeO_2_ NPs, Cf, and their composite material (CeO_2_/Cf) show different peaks (as shown in Fig. 1a). The spectrum of cerium oxide nanoparticles clearly shows three strong peaks at 3242, 1558 cm^−1^, and 700 cm^−1^. The large, broadband at 3242.6 cm^−1^ is due to the presence of phenolic compounds in ethanol extract (chlorogenic acid, catechol, ellagic acid, caffeic acid, gallic acid, protocatechin, coumarin, cinnamic acid, and ferulic acid) (Abdeldaiem and Hoda 2014). The weak absorption peaks at 2354 and 1430.7 cm^−1^ were because of the bending vibration of the C-H bonds of the methyl and methylene groups, respectively (Reddy et al. 2014). The absorption band close to 1634 cm^−1^ was because of the absorbed water molecule’s bending vibration, which can be observed in all samples (Farahmandjou et al. 2016; Su et al. 2017). The band at 1455 cm^−1^ matches to the C-H deformation of the methylene, methyl, and methoxy groups of lignin (Maheswari et al. 2012). The peak at 450–550 cm^−1^ is because of the O-Ce-O stretch vibration mode (McDevitt and Baun 1964).

However, in the case of Cf spectra (Fig. 1), the strength of the hemicellulose and lignin bands was reduced because of the destruction of the ester bonds of the carboxyl groups of the lignin and/or hemicellulose by chemical treatment. FTIR analysis confirmed the chemical composition of the sunflower seed extract and showed that the hemicellulose and lignin contents were significantly removed during the chemical treatment of the Cf extraction (Caschera et al. 2020; Adel et al. 2021; Al-shemy et al. 2022). The peaks observed in the wavenumber range of 3400–2920 cm^−1^ were characteristic of the stretching vibrations of the O-H and C-H groups of methyl and methylene in polysaccharides. The broad peak at 3400 cm^−1^ was characteristic of the hydroxyl stretching vibration in polysaccharides. This peak also includes intermolecular and intramolecular hydrogen bond vibrations in cellulose (Toro et al. 2020; El-Sabour et al. 2021). The band at 2894.8 cm^−1^ was because of the CH stretching vibration of all hydrocarbon components in polysaccharides (Reddy et al. 2018). The typical band of cellulose is observed in the region of 1650–900 cm^−1^, and the peak at 1647cm^−1^ corresponds to the vibration of water molecules absorbed by cellulose (Hospodarova et al. 2018). The small absorption band appears at 1456.8 cm^−1^, which corresponds to the CH strain in the lignin methoxyl group. The absorption bands at 1362, 1362, 1028 cm^−1^, and 895.4 cm^−1^ were associated with the C-O-C pyranose ring skeletal vibrations, C-O stretching, and C-H rocking vibrations from the cellulose, the band at 895.4 cm^−1^, due to the amorphous area in cellulose (Poletto et al. 2014). After cross-linking the reaction of Cf with CeO_2_ NPs (CeO_2_/Cf) at 80 °C for 24 h (Fig. 1A), it was observed that the (CeO_2_/Cf) nanocomposites show characteristic bands of CeO_2_ NPs and Cf.

However, a reduction in the band’s intensity was related to the hydroxyl group at 3400–3200 cm^−1^ compared to the pure Cf curve, which was measured by the absorbance ratio at 3286 cm^−1^, associated with the hydrogen bonding stretching vibration (OH…OH), and the reference band of β1-4 glycoside bond at 895.4 cm^−1^. The shift from 528 to 509 cm^−1^ of the characteristic peak of CeO_2_ NPs indicates interaction between Cf and cerium oxide in the sample (CeO_2_/Cf) (Ali et al. 2020). These outcomes indicate a chemical reaction between Cf and cerium oxide NPs through the hydroxyl and carboxyl groups of the fiber. The FTIR spectra of nanocomposites cross-linked with cerium titanate and cellulose fibers (0.1Ce-Ti/Cf, 0.3Ce-Ti/Cf, and 0.5Ce-Ti/Cf) in different molar ratios are presented in (Fig. 1B). Compared with the spectra of pristine cellulose fibers, the broadband presented at 3273 cm^−1^ because of the stretching mode of the O–H group, which illustrates water content in all samples (Ntwaeaborwa and Holloway 2005). The existence of cerium ions in Ce-Ti/Cf was clarified by the appearance of peaks at 541 cm^−1^ in all the materials of Ce-Ti/Cf nanocomposites, and this peak occurred because of Ce-O-Ce (Ge et al. 2007). The bending vibration of the Ti-O bond has a wide absorption peak at 668 cm^−1^, and light absorption at 510 cm^−1^ was because of the vibration of the Ti-O-Ti vibration stretching (Kong et al. 2018). The strength of these two absorption peaks was strengthened with an increase in molar ratios Ce-Ti, and the adsorption of water molecules on the surface of the samples Ce-Ti/Cf was improved. These findings show a strong interaction between Cf and Ce-Ti NPs through hydroxyl and carboxyl fiber groups.

**Fig. 2:**
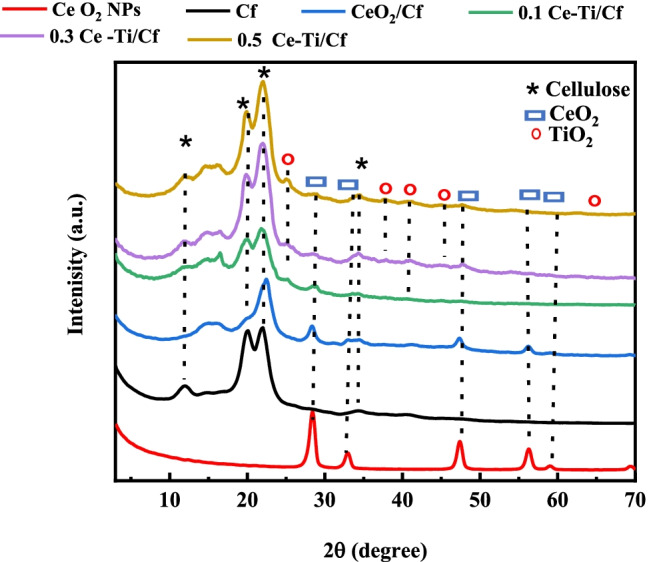
X-ray diffraction pattern of CeO_2_ NPs, Cf, CeO_2_/Cf, 0.1 Ce-Ti/Cf, 0.3 Ce-Ti/Cf, and 0.5 Ce-Ti/Cf nanocomposite materials

The XRD patterns of the pure Cf, CeO_2_ NPs, CeO_2_/Cf, and different concentrations of Ce-Ti-loaded cellulose fiber (0.1 Ce-Ti/Cf, 0.3 Ce-Ti/Cf, and 0.5 Ce-Ti/Cf) are presented in (Fig. 2). XRD analysis was used to characterize the fibers’ crystalline characteristics and determine the relationship between properties and fiber structures. The XRD spectrum (Fig. 2) of CeO_2_ NPs showed the typical diffraction peaks at 2θ = 28.38°, 32.9°, 47.31°, 56.26°, and 59.2°, which corresponded to (111), (200), (220), (311), and (222) atomic planes of CeO_2_ with cubic fluorite lattice structure, respectively (JCPDS card no. 34-0394) (French and Cintrón 2013; Adarakatti et al. 2018). The XRD pattern of Cf indicates that the Cf is semi-crystalline in character and showed diffraction peaks at 2θ = 11.9° (broad), 19.6° (sharp), 21.6° (sharp intense), and 34.8° (small) related to the (1–10 and 110), (020), and (004) crystallographic planes of the cellulose II, respectively (Reddy et al. 2018; Langan et al. 2021 ). After cross-linking reaction of Cf with CeO_2_ NPs (CeO_2_/Cf) at 80 °C for 24 h (Fig. 2), compared to the XRD curve for the control Cf, new peaks were detected in the curve of Cf. This is because of the diffraction peaks of the (111), (200), (220), (311), and (222) planes of CeO_2_ with the cubic fluorite structure (card no. 43-0394). It can also be found that the peaks of Cf (110) and (020) overlap each other and may be because of the formation of the nanostructures, lattice strain, and distortion (Agarwal et al. 2018). Furthermore, no peaks from impurities were observed in this figure; this clearly illustrates the high purity of all samples prepared. In addition to the diffraction peaks of cellulose fibers and CeO_2_ NPs, TiO_2_ anatase was detected in the curves of Ce-Ti loaded with cellulose fibers (0.1 Ce-Ti/Cf, 0.3 Ce-Ti/Cf, and 0.5 Ce-Ti/Cf) in different molar ratios (Fig. 2). However, TiO_2_ anatase diffraction peaks at 0.1 Ce-Ti/Cf and 0.3 Ce-Ti/Cf were detected in small amounts on photocatalytic cellulose, and this is because the low TiO_2_ content depends on total carbon and cellulose fibers. The strong diffraction peaks at 2θ =25.19° and 48.01° are also related to the (101) and (200) diffraction planes, respectively, indicating that the TiO_2_in the anatase phase is, according to card number 21-1272 (Chen and Mao 2007).

It is believed that in addition to the peaks at 0.1 Ce-Ti/Cf and 0.3 Ce-Ti/Cf, new peaks appeared at 0.5 Ce-Ti/Cf because of the high concentration of TiO_2_. Cross-linked TiO_2_ particles are utilized as effective positions to generate photogenerated electrons with sunlight, and then, Cf provides trapping sites for photogenerated electrons liberated from the titanium oxide conduction band. This should increase the composite fiber’s photocatalytic activity and expedite the breakdown of methylene blue and methyl orange (Zhang et al. 2013). The Segal formula calculates the crystallinity index (CI) of different cellulose samples and lists them in Table 1. The major crystalline peaks occur around 2θ = 22° and between 2θ values of 19° and 20°, respectively. The Segal crystallinity index ranged from 66.67% for Cf to 50.00% for 0.1 Ce-Ti/Cf. Whereas for Ce-Ti-loaded cellulose fiber (0.1 Ce-Ti/Cf, 0.3 Ce-Ti/Cf, and 0.5 Ce-Ti /Cf), the crystallinity index was 50.00%, 66.67, and 66.67%, respectively. These results indicate that incorporating NPs in CeO_2_ into Cf led to the rearrangement of cellulose fiber crystal planes, which may be because of higher hydrophilicity, and thus affinity to interact and contact with CeO_2_ NPs during the nanocomposite synthesis process. Shao et al. and Gutierrez et al. (Zhang et al. 2013; Gutierrez et al. 2013) reported that the incorporation of Ag NP and titanium/vanadium oxide, respectively, resulted in a significant reduction in the crystallinity index of bacterial cellulose (BC). However, according to this study’s findings, Erokh et al. (2016) observed that Cu_2_O nanoparticles synthesized on the surface of cotton fiber do not affect its morphology and structural characteristics.

**Table 1:** Relative crystallinity index (CrI) and d-spacing of Cf, CeO_2_/Cf, 0.1 Ce-Ti/Cf, 0.3 Ce-Ti/Cf, and 0.5 Ce-Ti/Cf nanocomposite materials

Material	d-spacing (nm)				CrI %
	(1–10)	(110)	(020)	(004)	
Cf	7.43	4.51	4.10	2.60	66.67
CeO_2_/Cf	6.16	4.43	3.93	2.58	65.75
0.1 Ce-Ti/Cf	7.13	4.41	4.03	2.65	50.00
0.3 Ce-Ti/Cf	7.46	4.44	4.05	2.61	66.67
0.5 Ce-Ti/Cf	7.49	4.51	4.03	2.60	66.67

These different impacts of many MNPs on the crystallinity of cellulose-based materials support our claim on the NPs affinity and support materials. Another observation in the XRD spectrum of the nanocomposite was the change of some diffraction peaks associated with Cfs; for example, the double diffraction peaks at 2θ of 19.6° and 21.6° were changed to a single diffraction peak. This change indicates that after interacting with the CeO_2_ NPs, the crystalline structure of Cf in some planes is affected, further confirming the production of real nanocomposites with strong interactions (Erokh et al. 2016). Table 1 presents a comparison of d-spacing and crystallinity index (CrI) before and after cross-linking reaction. Considering the d-spacing values for the cellulose polymorphs, CeO_2_/Cf presented a smaller d-spacing if compared with Cf.

**Fig. 3:**
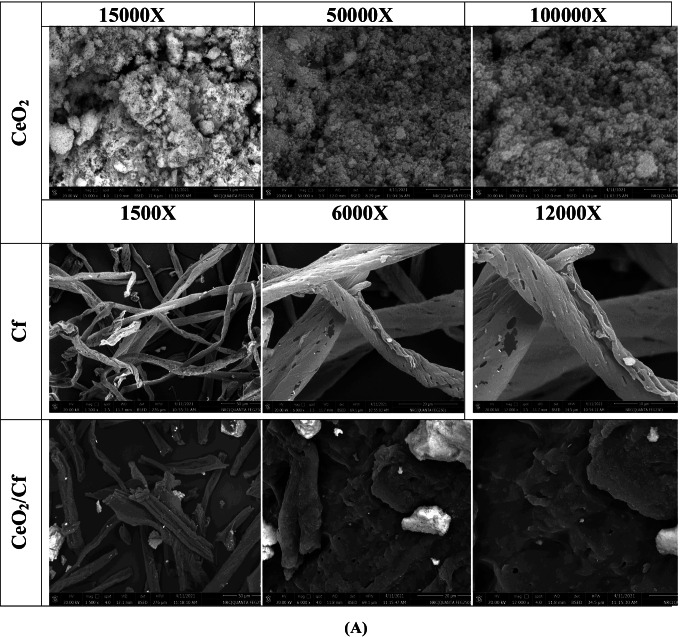

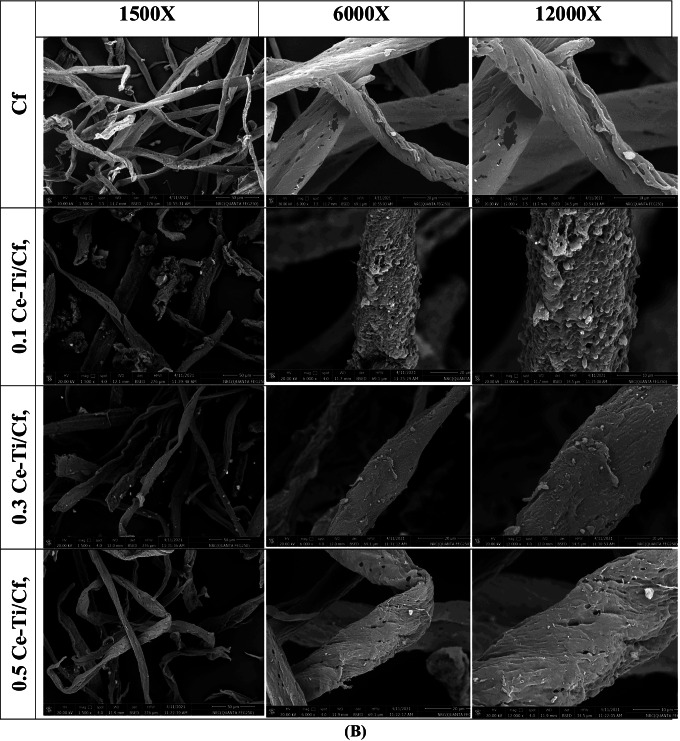
Scanning electron microscopy images of CeO_2_ NPs, Cf, and CeO_2_/Cf (**A**) and 0.1 Ce-Ti/Cf, 0.3 Ce-Ti/Cf, and 0.5 Ce-Ti/Cf nanocomposite materials (**B**)

**Fig. 4:**
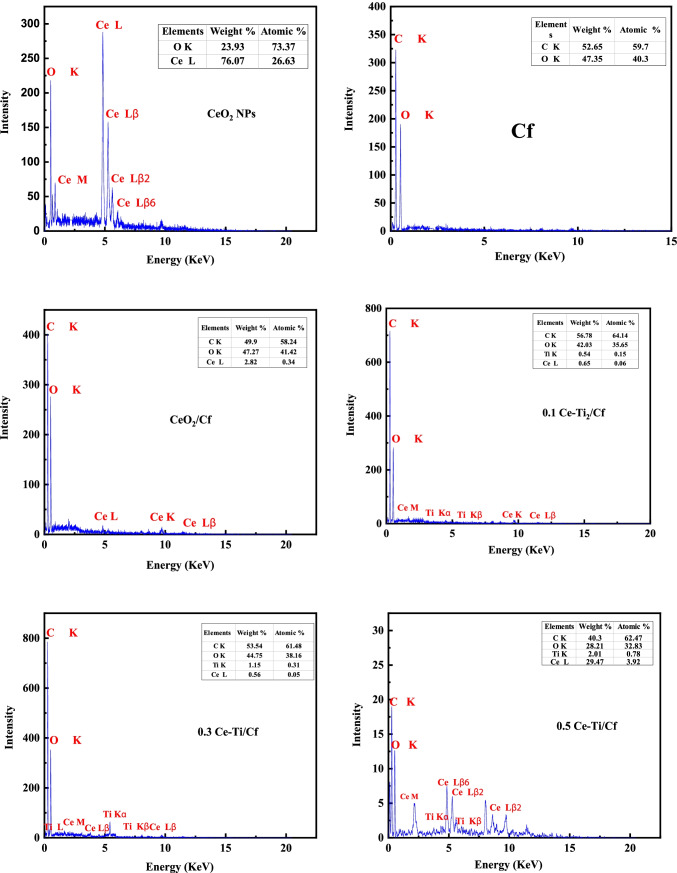
EDX analysis of CeO_2_ NPs, Cf, CeO_2_/Cf, 0.1 Ce-Ti/Cf, 0.3 Ce-Ti/Cf, and 0.5 Ce-Ti/Cf nanocomposite materials

A scanning electron microscope (SEM) technique was utilized to determine the morphologies of pure cellulose fiber (Cf), CeO_2_ NPs, (CeO_2_/Cf), and different molar ratios of Ce-Ti-loaded cellulose fiber (0.1 Ce-Ti/Cf, 0.3 Ce-Ti/Cf, and 0.5 Ce-Ti/Cf) as presented in Fig. 3. The detailed morphologies of CeO_2_NPs with higher magnifications showed coleus nanoparticles with asymmetrical shapes and a little number of spherical grains. The average particle size ranged from 26 to 61 nm Fig. 3a; also, EDXS spectra (Fig. 4) proved the existence of Ce and O elements in the CeO_2_NPs. It can be seen that the pure Cf presented in Fig. 3 appears to have a rough and clean surface with streaks because of the elimination of a great amount of hemicellulose closely related to cellulose and other substances by chemical treatment. Higher magnification shows that the surface of the original cellulose fibers is smooth, non-uniform with natural grooves and veins with cylindrical rod-like structures (Zhao et al. 2007). The EDX analysis confirmed that C and O were the merely elements that existed in the cellulose fiber, confirming the cellulose fiber host configuration. After cross-linking reaction of Cf with CeO_2_ NPs (CeO_2_/Cf) at 80 °C for 24 h, the micrographs were obtained using high magnification, exploring the morphology and cross-linkage of CeO_2_ with Cf. The micrographs show a coarse surface and porous structure because of the cross-linking attachment between CeO_2_NPs and the Cf polymer matrix, and there are visible points of aggregation. EDXS spectra established the existence of C, Ce, and O elements in the CeO_2_/Cf fiber. When the CeO_2_ NPs were cross-linked with the Cf, the CeO_2_ NP microspheres attached to Cf without destroying the inherent structure of the Cf. The same result was obtained when the incorporation of TiO_2_ was loaded on carbon fibers (Zhang et al. 2013; Wang et al. 2020)***.***

The summary of SEM images 0.1 Ce-Ti/Cf, 0.3 Ce-Ti/Cf, and 0.5 Ce-Ti/Cf nanocomposite photocatalyst illustrates a complex network configuration for these photocatalysts, and these fibers contained a lot of arbitrarily intersecting microfibers with high portion fractions. In addition, NPs from Ce-Ti are attached to the surface of Cf fibers as agglomerates and were unevenly distributed (fiber pieces produced through the cross-linking method) to make an extremely porous structure. Some of these nodes merge the arrangement. The entanglement connecting Ce-Ti NPs and Cf produces the mechanical features of nanocomposite fibers. EDXS analysis found that Ce-Ti/Cf fiber primarily consisted of O, Ti, C, and a small amount of Ce, titanium. In addition, heterogeneous accumulation of Ce-Ti was also observed in the area between adjacent fibers when the amount was increased to 0.5 Ce-Ti. The elemental analysis of pure Cf, CeO_2_ NPs, CeO_2_/Cf, and Ce-Ti-cross-linked cellulose fibers (0.1 Ce-Ti/Cf, 0.3 Ce-Ti/Cf, and 0.5 Ce-Ti/Cf) are presented in (Table 2). The elemental atomic composition of cellulose fiber (Cf) was 39.3 % C, 5.6 % H, and 0 % N. The N and C contents of cellulose fibers are the same as the N and C content of commercial cellulose (Biswal and Singh 2004); the elemental survey analysis of CeO_2_ NPs was 0.36 % C and 0.12% H. The observed contents of C and H for green synthesized CeO_2_ NPs were possibly observed because of the presence of few traces of plant extract or adsorption of organic impurities through synthesis (Sharma et al. 2017). When the CeO_2_ NPS were cross-linked with Cf, the C and N contents in (CeO_2_/Cf) were 38.7 % C, 5.27 % H, and 1.25 % N. The nitrogen content nanocomposite material (0.1 Ce-Ti/Cf, 0.3 Ce-Ti/Cf, and 0.5 Ce-Ti/Cf) was found to be 0.88, 1.5, and 2.4, respectively, which increases as the Ce-Ti molar ratio increases, as presented in (Table 2).

**Table 2:** Elemental analysis of the pure cellulose fiber (Cf), CeO_2_ NPs, CeO_2_/Cf, 0.1 Ce-Ti/Cf, 0.3 Ce-Ti/Cf, and 0.5 Ce-Ti/Cf nanocomposite materials

Materials	Elemental composition %			
	N	C	H	S
Cf	**0.00**	**39.30**	**5.60**	**0.00**
CeO_2_ NPs	**0.00**	**0.36**	**0.12**	**0.00**
CeO_2_/Cf	**1.25**	**38.70**	**5.27**	**0.00**
0.1 Ce-Ti/Cf	**0.88**	**42.30**	**6.40**	**0.00**
0.3 Ce-Ti/Cf	**1.50**	**41.50**	**5.70**	**0.00**
0.5 Ce-Ti/Cf	**2.40**	**40.10**	**5.70**	**0.00**

In addition, carbon content decreased to 42.3, 41.5, and 40.1, respectively. The considerable percentage of nitrogen in the cross-linked reaction accounted for the presence of cross-linked Ce-Ti chains; the variation in the nitrogen content, although not much, is significant in the case of 0.5 Ce-Ti/Cf. This is because of the higher Ce-Ti content in the nanocomposite polymer, which is also because of the higher number of moles of Ce-Ti in the reaction feed. Among other cross-linked polymers, 0.1 Ce-Ti/Cf has the lowest nitrogen content, when compared with other nanocomposites in this series (Gurung et al. 2014).

### Dye removal investigations

As illustrated in characterization tests, the synthesized Cf, CeO_2_ NPs, CeO_2_/Cf, and different concentrations of Ce-Ti-loaded cellulose fiber-containing different molar ratios of titanium (0.1 Ce-Ti/Cf, 0.3 Ce-Ti/Cf, and 0.5 Ce-Ti/Cf) nanocomposite photocatalyst have been successfully fabricated according to FE-SEM, FTIR, and XRD analyses. The photocatalytic degradation capabilities of the samples under sunlight illuminations were estimated through the degradation of methylene blue (MB) and methyl orange (MO), as model organic compounds are found in wastewaters:

#### Effects of dose on photocatalytic activity

**Fig. 5:**
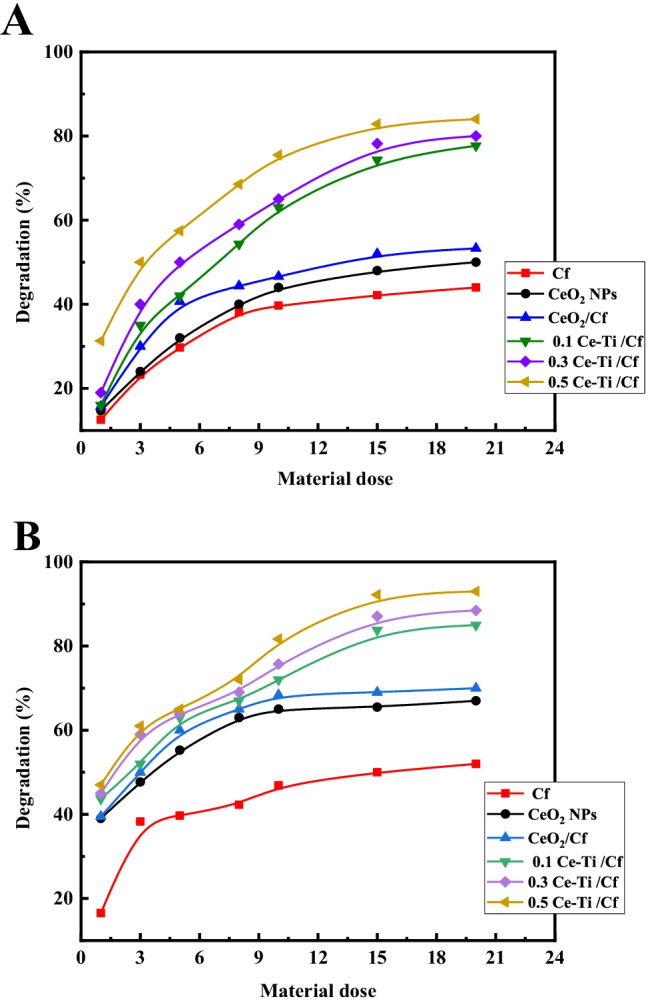
The photocatalytic efficiency of MB (**a**) and MO (**b**) by Ce-Ti /Cf nanocomposite materials with different molar ratios of titanate

Catalyst dosage affects the efficiency of photodegradation. Different Cf, CeO_2_ NPs, CeO_2_/Cf, 0.1 Ce-Ti/Cf, 0.3 Ce-Ti/Cf, and 0.5 Ce-Ti/Cf nanocomposite material doses in the range of 1–20 mg L^−1^ were dispersed into 20 mL solution of MO and MB at a concentration of 100 mg L^−1^. Before sunlight, adsorption equilibrium was reached by agitation at 120 rpm for 60 min in the dark. Figure 5 shows the relationship between the photodegradation rate of nanocomposite materials for raw Cf, CeO_2_ NPs, and CeO_2_/Cf, 0.1 Ce-Ti/Cf, 0.3 Ce-Ti/Cf, and 0.5 Ce-Ti/Cf doses.

As expected, the photodegradation efficiency increased with the catalyst dose because more binding sites were available for degradation. The reactivity of 0.1 Ce-Ti/Cf, 0.3 Ce-Ti/Cf, and 0.5 Ce-Ti/Cf at 20 mg L^−1^ was two times higher than raw Cf. However, the 15 mg L^−1^ dose showed the same good degradation behavior as 20 mg L^−1^, which is the reason behind its selection as the standard for subsequent experiments. Therefore, the nanocomposites 0.1 Ce-Ti/Cf, 0.3 Ce-Ti/Cf, and 0.5 Ce-Ti/Cf show the highest catalytic capacity to degrade MB and MO. The degradation rate of 0.5 Ce-Ti/Cf can degrade MB and MO by more than 85% and 93% in 5 h, respectively, indicating that active species that can degrade MB and MO dyes are easily produced during the activation process. However, when Ce-Ti nanoparticles are directly cross-linked with cellulose fibers, the rate of discoloration increases because of the presence of Ce-Ti nanoparticles in the manufacture of nanocomposites (Kotp 2020). Therefore, it can be concluded that Cf has a positive effect on the decoloration process. This increased decoloration can be because of the charge separation caused by the Ce-Ti charge on the Cf. For Cf with Ce-Ti, the main part of Cf is to transport electrons from Ce-Ti NPs, making it difficult to recombine electron pairs (Kotp 2020). Longer-lived electron-hole pairs led to the generation of a large number of photoreactive species.

Furthermore, because of the adsorption capacity of Cf and the porous structure of the nanocomposite, the MO and MB molecules are concentrated around Ce-Ti/Cf, which increases the probability that the MO and MB molecules diffuse and migrate to the exterior below a concentration slope. Therefore, the degradation percentage and the adequacy of MO and MB are correspondingly enhanced. As per the “Adsorb & Shuttle” (A&S) strategy, pollutants are adsorbed first and then dispersed to the TiO_2_ particles for degradation (Kedem et al. 2009).

Additionally, the loss of MO and MB is certainly attributable to photocatalytic degradation rather than adsorption. In all cases, there is still a little amount of MO and MB in the solution; those earnings that the remaining organic matter or intermediates are difficult to fully degrade because of the blocking of photocatalytic active sites in the process (An et al. 2012). Results show a notable difference in the degradation efficiency by 0.1 Ce-Ti/Cf, 0.3 Ce-Ti/Cf, 0.5 Ce-Ti/Cf, Cf, CeO_2_NPs, and CeO_2_/Cf. Therefore, 0.1 Ce-Ti/Cf, 0.3 Ce-Ti/Cf, and 0.5 Ce-Ti/Cf nanocomposite materials were used as the photocatalyst to investigate the removal of the MB and MO dyes in the following experiments, and the optimum dose is favored at 15 mg/L.

#### The effect of pH of MO and MB solutions on photocatalytic degradation

**Fig. 6:**
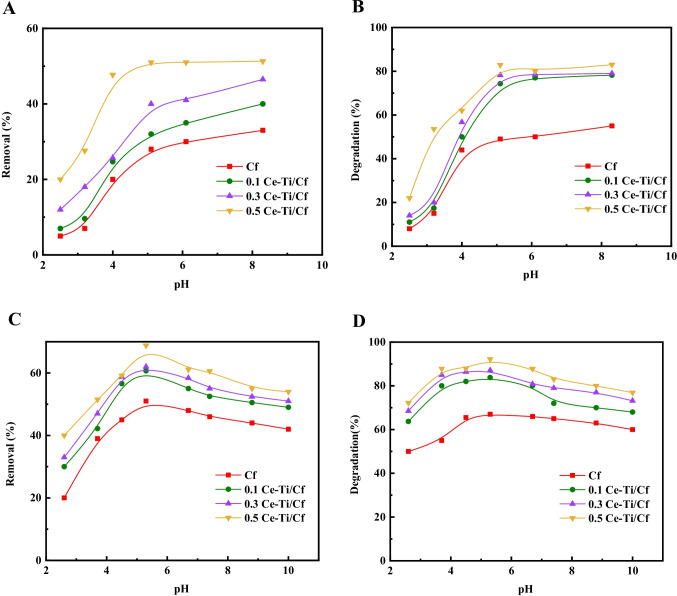
Influence of solution pH in the removal of MB and MO (**A**, **C**) and photodegradation of MB and MO (**B**, **D**) (10 mL 100 mg/L MB and MO, 0.15 g of Cf, 0.1 Ce-Ti/Cf, 0.3 Ce-Ti/Cf, and 0.5 Ce-Ti/Cf, reaction time: 3 h)

The pH value has a major impact on the exterior charge performance of the photocatalyst (Luo et al. 2015). In this experiment, sodium hydroxide and hydrochloric acid were utilized to adjust the pH of dye solutions. MO solution is orange in acidic condition and yellow in alkaline condition. The impact of change of the pH value from 2 to 10, and the initial dye concentration was 100 ppm, by 15 mg of Cf, 0.1 Ce-Ti/Cf, 0.3 Ce-Ti/Cf, and 0.5 Ce-Ti/Cf nanocomposite fibers in 20 mL solution of MB and MO for 3 h were plotted in (Fig. 6). The removal and degradation efficiency of MB rose with raising the pH value from acidic to alkaline direction (as shown in Fig. 6A, B). The 0.1 Ce-Ti/Cf, 0.3 Ce-Ti/Cf, and 0.5 Ce-Ti/Cf nanocomposite catalysts exhibited higher adsorption rates for MB (40, 46.5, and 51.3%), respectively, compared with Cf (33.1%) (Fig. 6 A), The abundant functional groups can explain the relatively higher adsorption rates of MB onto 0.1 Ce-Ti/Cf, 0.3 Ce-Ti/Cf, and 0.5 Ce-Ti/Cf, nanocomposite catalysts pore structure of Ce-Ti/Cf. After light irradiation, a photocatalytic reaction occurred, and the data indicated increased photocatalytic activity with increasing pH. The degradation ability of Cf was about 55.5 % within 3 h. After incorporating Ce-Ti, all 0.1 Ce-Ti/Cf, 0.3 Ce-Ti/Cf, and 0.5 Ce-Ti/Cf nanocomposite catalysts showed enhanced photodegradation of MB of 78.2, 79, and 83, respectively. The remaining concentration of MB after treatment with 0.5 Ce-Ti/Cf was approximately 17%, whereas, for Cf, it was approximately 44.5 % (Fig. 6 B). This high photocatalytic activity of Ce-Ti/Cf is due to its high adsorption capability for MB and the remarkable synergistic effect of the molecule interfacial layers (Farzana and Meenakshi 2014; Mohamed et al. 2015; Suet al. 2017). The same behavior has been stated previously by titania-silica/cobalt ferrite photocatalyst (Harraz et al. 2014). The pH value mainly affects the surface charge characteristics of the photocatalyst. In an alkaline solution, the surface of Ce-Ti can be negatively charged, while in an acid solution, it is positively charged (Zhang et al. 2018; Vieira et al. 2018). MB is a cationic dye, and it has a great tendency to be adsorbed onto the Cf, 0.1 Ce-Ti/Cf, 0.3 Ce-Ti/Cf, and 0.5 Ce-Ti/Cf surfaces at alkaline pH. This is because the highest degradation of 55, 78.2, 79, and 83 was achieved at pH 8.3, while a degradation efficiency of only 8, 11, 14, and 22% was obtained at acidic pH 2 for Cf, 0.1 Ce-Ti/Cf, 0.3 Ce-Ti/Cf, and 0.5 Ce-Ti/Cf, respectively.

Additionally, it is reasonable to suppose that the interaction between hydroxide ions (OH^−^) and photogenerated holes to create hydroxyl radicals (OH^−^) is improved at alkaline pH, which would assist photocatalytic degradation of MB (Harraz et al. 2014). As presented in (Fig. 6C, D), the reduction in pH significantly enhanced the photodegradation of 0.1 Ce-Ti/Cf, 0.3 Ce-Ti/Cf, and 0.5 Ce-Ti/Cf nanocomposites for MO. The improved adsorption at lower pH was due to the increased interaction connecting the protonated electrostatic MO and the negatively charged Cf or the sulfonic acid type of MO and the positively charged Ce-Ti NPs, which cross-linked with the Cf. As soon as the pH value is less than 4.00, the negative charge of the Cf is extensively declined because of reducing the separation of the carboxyl group, which leads to the reduction of the MO photodegradation (Andronic and Duta 2008). According to the work of the Andronic team (Andronic and Duta 2008), it can be seen that pH 5.3 is also conducive to MO photocatalytic reaction because of higher MO adsorption and the formation of azo structure in MO molecules. Then, choose the optimal pH value of 5.3 for the next experiment (Zhang et al. 2013; Kotp 2017). It can also be found in (Fig. 6) that the degradation efficiency of MB and MO increased in the order Cf ˂ 0.1 Ce-Ti/Cf ˂ 0.3 Ce-Ti/Cf ˂ 0.5 Ce-Ti/Cf. Therefore, 0.5 Ce-Ti/Cf nanocomposite materials showed highly efficient photocatalytic activity under sunlight irradiation due to the nano-scale effect of TiO_2_ anatase nanoparticles (Fig. 6 D). A further increase in the amount of TiO_2_ induced an increase in the photocatalytic MB and MO degradation (Wang et al. 2017).

#### Effect of degradation time

**Fig. 7:**
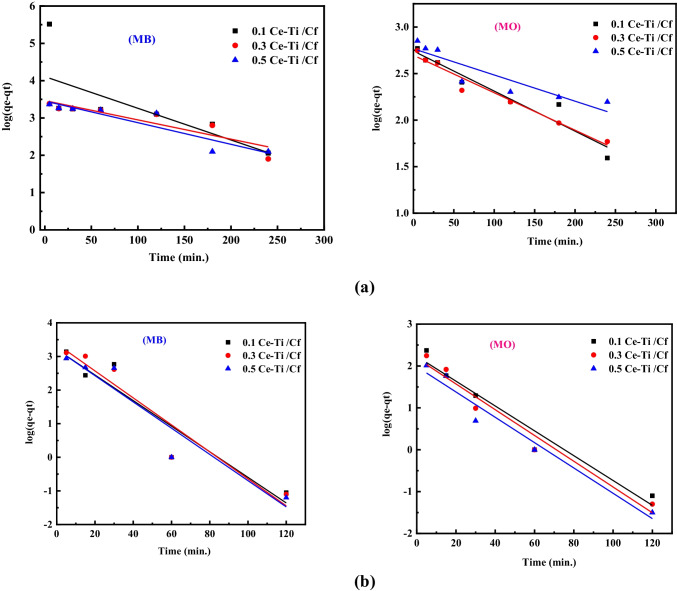
Linear fitting plots of a pseudo-first-order kinetic model for MB and MO photocatalytic degradation onto (0.1 Ce-Ti/Cf, 0.3 Ce-Ti/Cf, and 0.5 Ce-Ti/Cf nanocomposite materials in the absence (**a**) and presence of H_2_O_2_ (**b**)

**Fig. 8:**
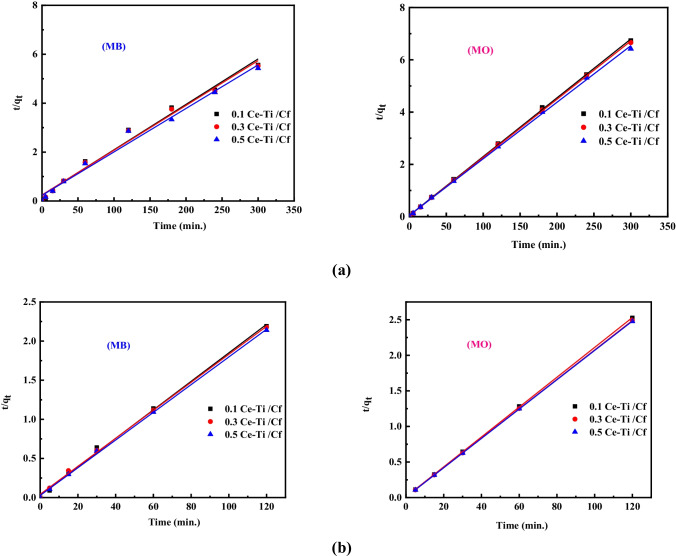
Linear fitting plots of a pseudo-second-order kinetic model for MB and MO photocatalytic degradation onto (0.1 Ce-Ti/Cf, 0.3 Ce-Ti/Cf, and 0.5 Ce-Ti/Cf) nanocomposite materials in the absence (**a**) and presence of H_2_O_2_ (**b**)

The impact of contact time on the degradation of MB and MO in the presence and absence of H_2_O_2_ by 0.1 Ce-Ti/Cf, 0.3 Ce-Ti/Cf, and 0.5 Ce-Ti/Cf nanocomposite materials at optimum pH and adsorbent dosage was examined. The findings of this investigation are summarized in (Figs. 7 and 8). The results indicate that the adsorption capacity and percentage of dye degradation increase significantly during the early contact time. They slow down after 120 min and achieve near equilibrium at about 240 min. As a result, the equilibrium period between dye and photocatalyst is optimized to 240 min. A comparison of degradation efficiency with and without H_2_O_2_ indicates that H_2_O_2_ enhances the degradation ability of the nanocomposite sites at the early interval times and reaches almost equilibrium at about 60 min, as presented in (Figs. 7 and 8).

The improvement of photodegradation of MB and MO with0.1 Ce-Ti/Cf, 0.3 Ce-Ti/Cf, and 0.5 Ce-Ti/Cf nanocomposite materials in the presence of H_2_O_2_ maybe because of the combined impacts of photodissociation of H_2_O_2_ with light and on the photocatalyst surface, and the complex transport of highly reactive hydroxyl radical (OH^●^) in the reactant mix. As per the literature (Barakat et al. 2005; Chiou et al. 2008), the next reaction schemes of the photolysis of H_2_O_2_ are possible:

3 $${\mathrm{H}}_2{\mathrm{O}}_2+ hv\to 2\mathrm{O}{\mathrm{H}}^{\bullet }$$

4 $$2{\mathrm{H}}_2{\mathrm{O}}_2\to 2{\mathrm{H}}_2\mathrm{O}+{\mathrm{O}}_2$$

5 $${\mathrm{e}}^{-}{\mathrm{H}}_2{\mathrm{O}}_2\to \mathrm{O}{\mathrm{H}}^{\bullet }+\mathrm{O}{\mathrm{H}}^{-}$$

6 $${h}^{+}\mathrm{OH}-\to \mathrm{O}{\mathrm{H}}^{\bullet }$$

7 $${\mathrm{e}}^{-}{\mathrm{O}}_2\to {\mathrm{O}}_2$$

8 $$\mathrm{TH}+\mathrm{O}{\mathrm{H}}^{\bullet }+{\mathrm{H}}_2\mathrm{O}+{\mathrm{R}}^{\bullet}\to \mathrm{further}\ \mathrm{oxidation}\ \left(\mathrm{TH}=\mathrm{organic}\ \mathrm{molecule}\right)$$

According to the reaction designs, the critical step is forming hydroxyl radicals via photolysis of H_2_O_2_, which can then be employed to react with organic compounds. H_2_O_2_ can potentially serve as an electron scavenger during photocatalysis. The hydroxide ion formed during the electron scavenging process of H_2_O_2_ may combine with holes (h^+^), thus decreasing electron-hole recombination in the photocatalyst’s molecular orbital (Chen et al. 1998; Yu et al. 2014). The superoxide ion (O_2_^−^) formed during the reaction can be employed to oxidize the organic molecule, but it is not as effective as the hydroxyl radical (Barakat et al. 2005). Along with photolysis, H_2_O_2_ may dissociate on the surface of the photocatalyst, resulting in the next reactions, as per Miller and Valentine (1999).

9 $${\mathrm{C}}^{+}+{\mathrm{H}}_2{\mathrm{O}}_2\to \mathrm{C}+{\mathrm{H}}^{+}+\mathrm{H}{{\mathrm{O}}_2}^{\bullet}\left({\mathrm{C}}^{+}=\mathrm{Oxidized}\ \mathrm{catalyzed}\ \mathrm{surface}\right)$$

10 $${\mathrm{C}}^{+}+{\mathrm{H}}_2{\mathrm{O}}_2\to {\mathrm{H}}^{-}+\mathrm{O}{\mathrm{H}}^{\bullet}\left(\mathrm{C}=\mathrm{Reduced}\ \mathrm{catalyzed}\ \mathrm{surface}\right)$$

11 $${\mathrm{C}}^{+}+{\mathrm{O}}_{2^{-}}\to \mathrm{C}+{\mathrm{O}}_2$$

12 $$\mathrm{C}+\mathrm{O}{{\mathrm{H}}^{\bullet}}_2\to {\mathrm{C}}^{+}+{\mathrm{O}}_{2^{-}}$$

13 $$\mathrm{C}+\mathrm{O}{\mathrm{H}}^{\bullet}\to {\mathrm{C}}^{+}+\mathrm{O}{\mathrm{H}}^{-}$$

Clearly, H_2_O_2_ can react with both reduced and oxidized catalyst surfaces in these model equations, and the catalyst and H_2_O_2_ can conduct concurrent redox reactions. These reactions create extra hydroxyl radicals and ions, serving as electron/hole scavengers and reacting with organic compounds. As demonstrated in the reaction (11)–(13), hydroxyl, superoxide, and per hydroxyl radials could additionally react on the catalyst surface, thereby cumulatively enhancing the photocatalytic reaction. This set of reactions may explain the increase in the overall photodegradation rate of methylene blue and methyl orange in 0.1 Ce-Ti/Cf, 0.3 Ce-Ti/Cf, and 0.5 Ce-Ti/Cf nanocomposite materials. To explore, the degradation reaction kinetics of MB and MO by 0.1 Ce-Ti/Cf, 0.3 Ce-Ti/Cf, and 0.5 Ce-Ti/Cf were used in the present work, pseudo-first-order (Eq. (14)), and pseudo-second-order (Eq. (15)), through the application of linearized equations:

14 $$\log \left(\mathrm{qe}-\mathrm{qt}\right)=\log qe-\frac{K1t}{2.303}$$

15 $$\frac{t}{qt}=\frac{1}{K2\times qe2}+\frac{t}{qe}$$

where qt and qe (mg/g) denote the dye’s adsorption capacity at time *t* and equilibrium, respectively, and k1 (min^−1^) and k2 (g/(mg min)) denote pseudo-first- and pseudo-second-order efficiency constants, respectively. The kinetic factors adsorption capacity, rate constant, and correlation coefficient (*R*^2^) were calculated from linear plots of lg (qe-qt) versus time for the pseudo-first-order model and t/qt overtime for the pseudo-second-order model (as shown in Tables 3 and 4). Following a comparison of the correlation coefficients for pseudo-first- and pseudo-second-order (presented in Fig. 8), it is clear that the adsorption behavior of MB and MO onto 0.1 Ce-Ti/Cf, 0.3 Ce-Ti/Cf, and 0.5 Ce-Ti/Cf nanocomposite materials follow pseudo-second-order kinetics (*R*^2^ > 0.99) rather than first order. The *R*^2^ value for the pseudo-second-order model was greater compared to the pseudo-first-order model.

**Table 3:** Photodegradation kinetic parameters for the MB and MO degradation without H_2_O_2_ addition

	Pseudo-first-order model							
	MB				MO			
Materials	q_e_,exp (mg/g)	K_1_ (min^−1^)	q_e_,cal (mg/g)	*R*^2^	q_e_,exp (mg/g)	K_1_ (min^−1^)	q_e_,cal (mg/g)	*R*^2^
0.1 Ce-Ti/Cf	50.50	0.019	12.82	0.52	44.56	0.0090	55.08	0.93
0.3 Ce-Ti/Cf	53.92	0.115	28.84	0.82	45.05	0.0092	48.97	0.96
0.5 Ce-Ti/Cf	54.29	0.133	28.98	0.86	46.71	0.0064	57.54	0.83
	Pseudo-second-order model							
Materials	MB				MO			
	K_2_ (g/mg min)	q_e_,cal (mg/g)	*R*^2^		K_2_ (g/mg min)	q_e_,cal (mg/g)	*R*^2^	
0.1 Ce-Ti/Cf	0.002	55.55	0.98		0.009	45.45	0.99	
0.3 Ce-Ti/Cf	0.003	54.64	0.98		0.011	45.45	0.99	
0.5 Ce-Ti/Cf	0.003	58.82	0.98		0.128	49.23	0.99

**Table 4:** Photodegradation kinetic parameters for the MB and MO degradation in the presence of H_2_O_2_

	Pseudo-first-order model							
	MB				MO			
Materials	q_e_,exp (mg/g)	K_1_ (min^−1^)	q_e_,cal (mg/g)	*R*^2^	q_e_,exp (mg/g)	K_1_ (min^−1^)	q_e_,cal (mg/g)	*R*^2^
0.1 Ce-Ti/Cf	52.67	0.087	162.18	0.96	46.76	0.066	169.8244	0.89
0.3 Ce-Ti/Cf	53. 69	0.092	234.42	0.96	47.94	0.069	151.3561	0.91
0.5 Ce-Ti/Cf	54.88	0.089	158.42	0.96	48.09	0.069	95.49926	0.91
	Pseudo-second-order model							
Materials	MB				MO			
	K_2_ (g/mg min)	q_e_,cal (mg/g)	*R*^2^		K_2_ (g/mg min)	q_e_,cal (mg/g)	*R*^2^	
0.1 Ce-Ti/Cf	0.011	54.94	0.99		0.033	50.00	0.99	
0.3 Ce-Ti/Cf	0.007	56.17	0.99		0.054	58.54	0.99	
0.5 Ce-Ti/Cf	0.011	58.49	0.99		0.060	59.54	0.99	

Furthermore, the experimental qe value (qe, exp) was more consistent with the estimated values (qe, cal) derived from pseudo-second-order kinetics. Thus, these considerations show that the degradation of MB and MO is a chemical process, owing to the considerable role of chemical bonding and electron transfer. Additionally, the rate-limiting step could be a chemical reaction, and intra-particle diffusion could be involved in the process (Tajizadegan et al. 2015; Zhai et al. 2018). Furthermore, all samples showed excellent photocatalytic activity for the degradation of MO and MB, reaching approximately 95.4 and 92.7, respectively, for 0.5 Ce-Ti/Cf within 300 min under sunlight. Meanwhile, the photodegradation rates for 0.1 Ce-Ti/Cf (91.12%, 90.39%) and 0.3 Ce-Ti/Cf (92%, 91%) for MO and MB, respectively. These results can be because of the higher molar ratio of Ce-Ti contents of the Ce-Ti/Cf nanocomposite fibers with stronger degradation properties according to EDXS spectra. Based on the results presented above, sample 0.5 Ce-Ti/Cf prepared using 0.5 M Ce-Ti NPs and a reduction time of 300 min was chosen to investigate representative material performance further.

### Photocatalytic mechanism

**Fig. 9:**
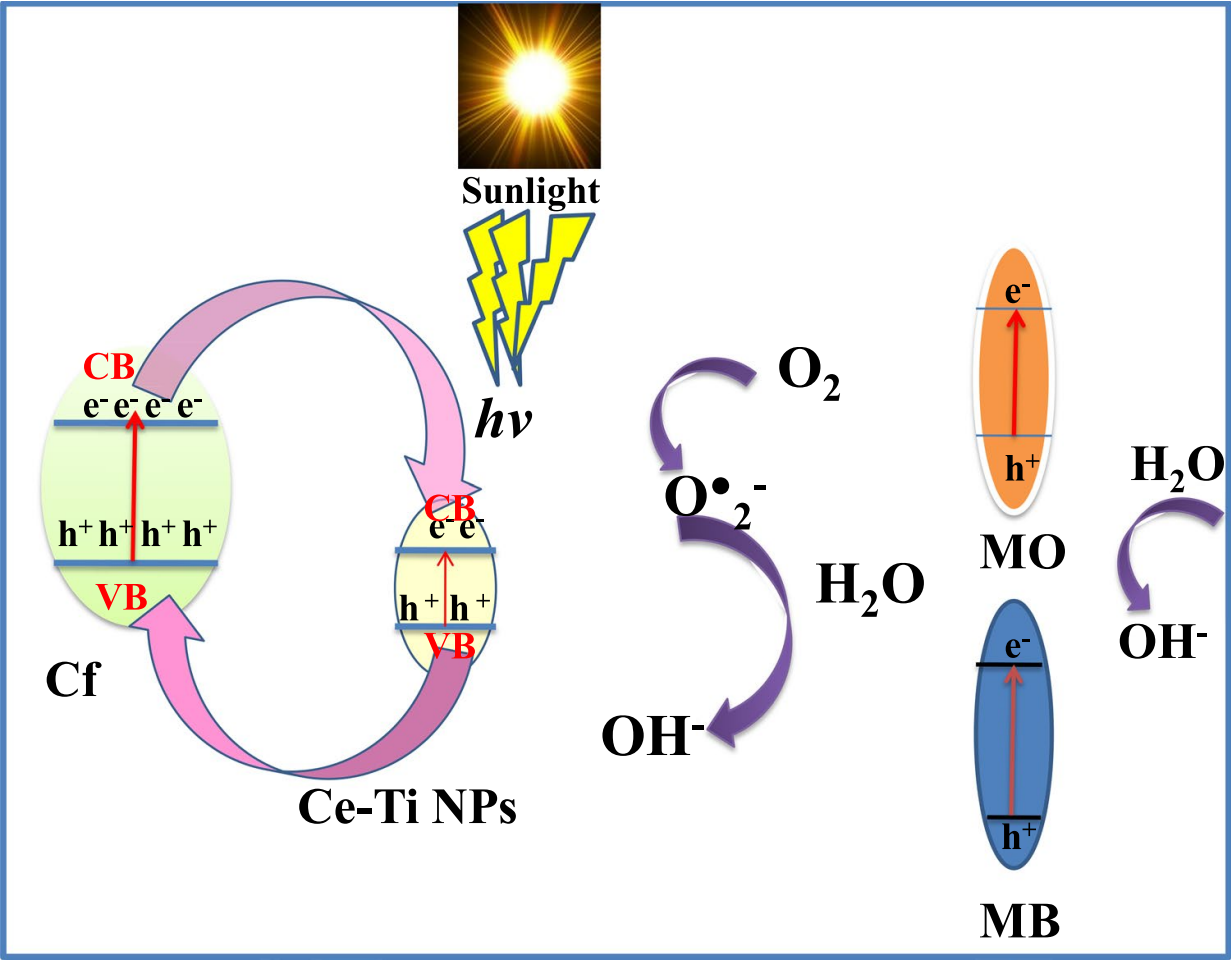
Proposed mechanism of Ce-Ti/Cf nanocomposite photocatalysis for the generation of different active species under sunlight illumination

The increased photocatalytic activity of the 0.1 Ce-Ti/Cf, 0.3 Ce-Ti/Cf, and 0.5 Ce-Ti/Cf nanocomposite materials can be attributed to the increased charge separation caused by the integration of varying molar ratios of Ce-Ti NPs into the Cf. It is well established that the photocatalyst’s photogenerated electron-hole separation efficiency is proportional to its quantum efficiency and light energy utilization ratio. As illustrated in Fig. 9, when the Ce-Ti/Cf nanocomposite photocatalyst is exposed to sunlight, cross-linked Cf is easily excited, generating photo-induced holes and electrons. The band structures of cross-linked Cf and Ce-Ti were found to be quite similar, with Cf having a greater conduction band edge (CB) than Ce-Ti and Ce-Ti having a lower valence band edge (VB) than Cf (Abdel-galil et al. 2014). As a result, some of the excited electrons in the CB of Cf migrate to the CB of Ce-Ti with lower energy, while some of the photogenerated holes in the VB of Ce-Ti migrate to the VB of Cf, aiding in charge separation. Because the energy of the VB of Cf is greater than that of Ce-Ti, additional holes in the VB of Cf were produced as a result of electron transfer to the VB of Ce-Ti. The electrons transferred from Cf to the VB of Ce-Ti diminish the quantity of holes in Ce-Ti. This decreases the recombination rate and increases charge separation in the composite’s VB and CB, which is necessary to increase photocatalytic activity; this is an advantage of employing a linked system. Additionally, the optical band gap changes from indirect to direct when various molar ratios of Ce-Ti to Cf are added, owing to the increase in TiO_2_ concentration. This phenomenon is caused by the transition from an amorphous to a nanocrystalline structure.

Furthermore, E_opt_ (optical band gap) decreases with increasing TiO_2_ content (Ali et al. 2019). This is highlighted by the fact that the band gap of semiconductors is particle size dependent (Reddy et al. 2001). The band gap narrows as particle size increases, and the absorption edge shifts toward lower energy as particle size increases. This effect fits with the rise of crystallinity with the rise of the Ce-Ti molar ratio; MB was also excited under sunlight irradiation to MB*, followed by photo-induced electron transfer from MB* to CB of Ce-Ti, that react with adsorbed species, often O_2_, to generate reactive superoxide radical anion. As the CB of Ce-Ti and Cf lies above the reduction potential of O_2_/O_2_^•−^ (superoxide radical anion), O^•^_2_^−^ was created by combining electrons from CB with the dissolved oxygen. The reaction of holes with H_2_O forms the hydroxyl radicals (OH•). The hydroxyl radicals effectively decompose the organic pollutant MB. On the basis of the experimental results and comments above, a photocatalytic degradation mechanism for MB and MO dyes on the 0.5 Ce-Ti/Cf nanocomposite catalyst may be postulated, as detailed in Eqs. (16)–(28) (Chatterjee et al. 2019).

16 $$\mathrm{Cf}+ hv\left({{\mathrm{h}}^{+}}_{\mathrm{VB}}+{{\mathrm{e}}^{-}}_{\mathrm{CB}}\right)\mathrm{Cf}$$

17 $$0.5\ \mathrm{Ce}-\mathrm{Ti}+ hv\to \left({{\mathrm{h}}^{+}}_{\mathrm{VB}}+{{\mathrm{e}}^{-}}_{\mathrm{CB}}\right)\mathrm{Ce}-\mathrm{Ti}$$

18 $$\left({{\mathrm{e}}^{-}}_{\mathrm{CB}}\right)\mathrm{Ce}-\mathrm{Ti}+{\mathrm{O}}_2\to {\mathrm{O}}_{2^{\bullet -}}$$

19 $${\mathrm{H}}_2\mathrm{O}+{\mathrm{O}}_{2^{\bullet -}}\to {}^{\bullet}\mathrm{OOH}+\mathrm{O}{\mathrm{H}}^{-}$$

20 $${}^{\bullet}\mathrm{OOH}+{\mathrm{H}}_2\mathrm{O}\to {}^{\bullet}\mathrm{OH}+{\mathrm{H}}_2{\mathrm{O}}_2$$

21 $${\mathrm{H}}_2{\mathrm{O}}_2+\left({\mathrm{e}}^{-}\right)\mathrm{Ce}-\mathrm{Ti}\to {}^{\bullet}\mathrm{OH}+\mathrm{O}{\mathrm{H}}^{-}$$

22 $${\mathrm{H}}_2{\mathrm{O}}_2+\left({{\mathrm{h}}^{+}}_{\mathrm{VB}}\right)\ \mathrm{Cf}\to \mathrm{OOH}+{\mathrm{H}}^{+}$$

23 $${\mathrm{H}}_2{\mathrm{O}}_2+{}^{\bullet}\mathrm{OOH}\to {}^{\bullet}\mathrm{OH}+{\mathrm{H}}_2\mathrm{O}+{\mathrm{O}}_2$$

24 $$\mathrm{Dye}+ hv\to {\mathrm{Dye}}^{\bullet }$$

25 $${\mathrm{Dye}}^{\bullet}\to {\mathrm{Dye}}^{+}+\left({{\mathrm{e}}^{-}}_{\mathrm{CB}}\right)\ \mathrm{Ce}-\mathrm{Ti}/\mathrm{Cf}$$

26 $${\mathrm{Dye}}^{\bullet }+\left({{\mathrm{h}}^{+}}_{\mathrm{VB}}\right)\ \mathrm{Cf}\to {\mathrm{Dye}}^{+}$$

27 $$\left({{\mathrm{e}}^{-}}_{\mathrm{CB}}\right)\ \mathrm{Ce}-\mathrm{Ti}/\mathrm{Cf}+{\mathrm{O}}_2\to {{\mathrm{O}}^{\bullet}}_{2^{-}}$$

28 $${}^{\bullet}\mathrm{OH}+{{\mathrm{O}}^{\bullet}}_{2^{-}}+{\mathrm{Dye}}^{+}\to \mathrm{Colorless}\ \mathrm{degraded}\ \mathrm{products}$$

### Applications of sunlight energy for real wastewater treatment using 0.1 Ce-Ti/Cf, 0.3 Ce-Ti/Cf, and 0.5 Ce-Ti/Cf nanocomposite materials

Highly active (0.1 Ce-Ti/Cf, 0.3 Ce-Ti/Cf, and 0.5 Ce-Ti/Cf) nanocomposite materials were applied to degrade real textile factory wastewater (RWW) on the 10th of Ramadan City, Egypt. The results were collected in Table 5. The COD value of RWW following photocatalytic treatment with green-produced nanocomposite materials was determined in this work using the open reflux method (Method no. 5220B) (APHA 2012). The COD result for untreated RWW (control) was 1200 mg L^−1^, which was significantly above than the allowed limit (120 mg L^−1^). RWW’s high COD value could result from its high organic loading and recalcitrant organic contaminants. RWW with a high COD value disrupts the ecological functioning of receiving water bodies, affecting aquatic life (Goutam et al. 2018). Nevertheless, the elimination in COD was obtained after the sunlight photocatalytic treatment of RWW with green synthesized nanocomposite materials, and the removal efficiency was 56.6, 64.1, and 90.8 % for 0.1 Ce-Ti/Cf, 0.3 Ce-Ti/Cf, and 0.5 Ce-Ti/Cf nanocomposite materials, respectively. The results of photodegradation are illustrated in (Table 5), and the percentage removal of COD from RWW was measured as per Eq. (2).

**Table 5:** Physico-chemical characteristics of treated real wastewater

Analytical parameter	Water samples results (wastewater (mg/L))	Product water results		
		0.1 Ce-Ti/Cf (mg/L)	0.3 Ce-Ti/Cf (mg/L)	0.5 Ce-Ti/Cf (mg/L)
TDS	5457.52	1575.37	912.82	662.61
PH	7.80	7.60	7.50	7.00
COD	1200.00	520.00	430	110.00
Phenol	5.50	0.89	0.47	0.27
MO	10.70	2.28	1.76	1.02
MB	2.75	0.00	0.00	0.00
Congo red	30.10	4.80	3.60	2.30.00
Malachite green	7.29	0.40	0.40	0.30
Safranin	8.68	0.48	0.45	0.42
SO_4_	48.27	19.14	18.69	15.60
Al	1.50	1.10	0.30	0.17
Ba	0.31	0.03	0.02	0.015
Cu	0.42	0.14	0.12	0.12
Si	3.3.00	2.3.00	2.2.00	1.2.00
Sr	0.10	0.002	0.002	0.001

The statistical analysis results are shown in Tables 6 and 7. The sum of mean squares, *p*-values, and *F*-values are all common aspects of ANOVA. These values indicate whether or not the model is statistically valid. As shown in the tables, the *F*-values were more than the *p*-values for each term in the model for all nanocomposite materials, suggesting the relevance of each interaction term. The *F*-value probability happening because of noise was 0.01% in each case. Furthermore, the *p*-values for all model terms were less than 0.05, which presented the significance of these model terms (COD, phenols, MB, MO, and sulfate) and can therefore be generalized to a broader range of interest for degradation in the real textile wastewater (RWW) sample against 0.1 Ce-Ti/Cf, 0.3 Ce-Ti/Cf, and 0.5 Ce-Ti/Cf nanocomposite materials. The degradation efficiency achievements of 0.1 Ce-Ti/Cf, 0.3 Ce-Ti/Cf, and 0.5 Ce-Ti/Cf nanocomposite materials toward MB and MO were compared to other reported functional fabrics as exposed in Table 8. 0.5 Ce-Ti/Cf nanocomposite materials showed better than other reported catalysts. So, an effective degradation process and ease of preparation of 0.5 Ce-Ti nanoparticles allow 0.5 Ce-Ti/Cf nanocomposite photocatalyst to be a promising photocatalyst utilized in treating different wastewater in Egypt.

**Table 6:** Analysis of variance (ANOVA) for the fit of degradation efficiency of COD, phenol, MO, MB, and SO_4_ by 0.1 Ce-Ti/Cf, 0.3 Ce-Ti/Cf, and 0.5 Ce-Ti/Cf nanocomposite materials

ANOVA							
		Sum of squares	Dif.	Mean square	F	Sig.	
COD	Between groups	1954.182	2	977.091	47,966.296	.000	Significant
	Within groups	.122	6	.020			
	Total	1954.305	8				
Phenol	Between groups	201.492	2	100.746	14,304.078	.000	
	Within groups	.042	6	.007			
	Total	201.534	8				
MO	Between groups	208.127	2	104.063	9361.538	.000	
	Within groups	.067	6	.011			
	Total	208.194	8				
MB	Between groups	1.870	2	.935	9349.000	.000	
	Within groups	.001	6	.000			
	Total	1.870	8				
SO_4_	Between groups	94.527	2	47.264	3212.174	.000	
	Within groups	.088	6	.015			
	Total	94.615	8

**Table 7:** LSD of different 0.1 Ce-Ti/Cf, 0.3 Ce-Ti/Cf, and 0.5 Ce-Ti/Cf nanocomposite materials, which applied to the real wastewater

Multiple comparisons							
LSD							
Dependent variable	(I) nanocomposite materials	(J) nanocomposite materials	Mean difference (I-J)	Std. error	Sig.	95% confidence interval	
						Lower bound	Upper bound
COD	**0.1 Ce-Ti/Cf**	**0.1 Ce-Ti/Cf**					
		**0.3 Ce-Ti/Cf**	− 7.70000^*^	.11653	.000	− 7.9851	− 7.4149
		**0.5 Ce-Ti/Cf**	− 34.38889^*^	.11653	.000	− 34.6740	− 34.1037
	**0.3 Ce-Ti/Cf**	**0.1 Ce-Ti/Cf**	7.70000^*^	.11653	.000	7.4149	7.9851
		**0.3 Ce-Ti/Cf**					
		**0.5 Ce-Ti/Cf**	− 26.68889^*^	.11653	.000	− 26.9740	− 26.4037
	**0.5 Ce-Ti/Cf**	**0.1 Ce-Ti/Cf**	34.38889^*^	.11653	.000	34.1037	34.6740
		**0.3 Ce-Ti/Cf**	26.68889^*^	.11653	.000	26.4037	26.9740
		**0.5 Ce-Ti/Cf**					
Phenol	**0.1 Ce-Ti/Cf**	**0.1 Ce-Ti/Cf**					
		**0.3 Ce-Ti/Cf**	− 7.67879-^*^	.06852	.000	− 7.8465	− 7.5111
		**0.5 Ce-Ti/Cf**	− 11.35758^*^	.06852	.000	− 11.5252	− 11.1899
	**0.3 Ce-Ti/Cf**	**0.1 Ce-Ti/Cf**	7.67879^*^	.06852	.000	7.5111	7.8465
		**0.3 Ce-Ti/Cf**					
		**0.5 Ce-Ti/Cf**	− 3.67879^*^	.06852	.000	− 3.8465	− 3.5111
	**0.5 Ce-Ti/Cf**	**0.1 Ce-Ti/Cf**	11.35758^*^	.06852	.000	11.1899	11.5252
		**0.3 Ce-Ti/Cf**	3.67879^*^	.06852	.000	3.5111	3.8465
		**0.5 Ce-Ti/Cf**					
MO	**0.1 Ce-Ti/Cf**	**0.1 Ce-Ti/Cf**					
		**0.3 Ce-Ti/Cf**	− 4.88660^*^	.08609	.000	− 5.0972	− 4.6760
		**0.5 Ce-Ti/Cf**	− 11.72523^*^	.08609	.000	− 11.9359	− 11.5146
	**0.3 Ce-Ti/Cf**	**0.1 Ce-Ti/Cf**	4.88660^*^	.08609	.000	4.6760	5.0972
		**0.3 Ce-Ti/Cf**					
		**0.5 Ce-Ti/Cf**	− 6.83863^*^	.08609	.000	− 7.0493	− 6.6280-
	**0.5 Ce-Ti/Cf**	**0.1 Ce-Ti/Cf**	11.72523^*^	.08609	.000	11.5146	11.9359
		**0.3 Ce-Ti/Cf**	6.83863^*^	.08609	.000	6.6280	7.0493
		**0.5 Ce-Ti/Cf**					
MB	**0.1 Ce-Ti/Cf**	**0.1 Ce-Ti/Cf**					
		**0.3 Ce-Ti/Cf**	− .93000^*^	.00816	.000	− .9500	− .9100
		**0.5 Ce-Ti/Cf**	− 1.00000^*^	.00816	.000	− 1.0200	− .9800
	**0.3 Ce-Ti/Cf**	**0.1 Ce-Ti/Cf**	.93000^*^	.00816	.000	.9100	.9500
		**0.3 Ce-Ti/Cf**					
		**0.5 Ce-Ti/Cf**	− .07000^*^	.00816	.000	− .0900	− .0500
	**0.5 Ce-Ti/Cf**	**0.1 Ce-Ti/Cf**	1.00000^*^	.00816	.000	.9800	1.0200
		**0.3 Ce-Ti/Cf**	.07000^*^	.00816	.000	.0500	.0900
		**0.5 Ce-Ti/Cf**					
SO_4_	**0.1 Ce-Ti/Cf**	**0.1 Ce-Ti/Cf**					
		**0.3 Ce-Ti/Cf**	− .97742^*^	.09904	.000	− 1.2198	− .7351
		**0.5 Ce-Ti/Cf**	− 7.31125^*^	.09904	.000	− 7.5536	− 7.0689
	**0.3 Ce-Ti/Cf**	**0.1 Ce-Ti/Cf**	.97742^*^	.09904	.000	.7351	1.2198
		**0.3 Ce-Ti/Cf**					
		**0.5 Ce-Ti/Cf**	− 6.33383^*^	.09904	.000	− 6.5762	− 6.0915
	**0.5 Ce-Ti/Cf**	**0.1 Ce-Ti/Cf**	7.31125^*^	.09904	.000	7.0689	7.5536
		**0.3 Ce-Ti/Cf**	6.33383^*^	.09904	.000	6.0915	6.5762
		**0.5 Ce-Ti/Cf**					

*The mean difference is significant at the 0.05 level

**Table 8:** Examples of photocatalytic cellulose materials for degradation of MB and MO in recent years

Catalyst type	Pollutant	Performance	References
Ag/ZnO cotton fabric	MB, 20 mg L^−1^, 40 mL	120 min, 90%, UV–vis	Ibanescu et al. (2014)
GO/TiO_2_ cotton fabric	MB, 10 mg L^−1^, 100 mL	4 days, 90%, visible	Karimi et al. (2016)
TiO_2_ cotton fabric	MB, 10 mg L^−1^, 100 mL	14 h, 90%, UV–vis	Cheng et al. (2018)
Ag/AgCl/ZIF-8/TiO_2_ cotton fabric	MB, 20 mg L^−1^, 50 mL	105 min, 98.5%, visible	Guan et al. (2019)
Ag@AgCl-reinforced cellulose composites	MB, 15 mg L^−1^, 50 mL	60min, 99%, UV–vis	Dong et al. (2021)
0.1Ce-Ti/Cf	MB, 100 mg L^−1^, 10 mL	120 min, 91.8%, sunlight	This work
0.3 Ce-Ti/Cf	MB, 100 mg L^−1^, 10 mL	120 min, 92.3%, sunlight	This work
0.5 Ce-Ti/Cf	MB, 100 mg L^−1^, 10 mL	120 min, 94%, sunlight	This work
C-T/CA fiber	MO, 40 mg L^−1^, 20 mL	60min, 98%, UV–vis	Shi et al. (2019)
CA/ZnO -0.4	MO, 20 mg L^−1^, 100 mL	90min, 94.71%, UV–vis	Hasanpour et al. (2021)
H_4_SiW_12_O_40_ (SiW_12_)/cellulose acetate (CA)	MO, 10 mg L^−1^, 100 mL	120min, 94.6%, UV–vis	Li et al. (2017)
TiO_2_ templated by NFC	MO, 5 mg L^−1^, 100 mL	120min, 100%, UV–vis	Xiao et al. (2017)
BC/PDA/TiO_2_ fabric	MO, 20 mg L^−1^, 20 mL	30min, 95.1%, UV–vis	Yang et al. (2020)
TiO_2_/cellulose fabric	MO, 30 mg L^−1^, 25 mL	100min, 99.5%, UV–vis	Chu et al. (2019)
0.1Ce-Ti/Cf	MO, 100 mg L^−1^, 10 mL	60 min, 97%, sunlight	This work
0.3 Ce-Ti/Cf	MO, 100 mg L^−1^, 10 mL	60 min, 98.5%, sunlight	This work
0.5 Ce-Ti/Cf	MO, 100 mg L^−1^, 10 mL	60 min, 98.9%, sunlight	This work

## Conclusion

In this work, 0.1 Ce-Ti/Cf, 0.3 Ce-Ti/Cf, and 0.5 Ce-Ti/Cf nanocomposite as a highly efficient photocatalyst for the degradation of organic dyes has been successfully developed for the first time by using the green synthesis technique. A combination of two semiconductors (CeO_2_ and TiO_2_) could induce mutual properties that would improve the activity of the hybrid nanocomposites compared to the individual materials (Cf and CeO_2_ NPs). The fabricated nanocomposites (0.1 Ce-Ti/Cf, 0.3 Ce-Ti/Cf, and 0.5 Ce-Ti/Cf) were described by FTIR, SEM, XRD, and elemental analysis measurement, respectively, to obtain their chemical and morphological structures. Also, the photodegradation efficiency of methylene blue (MB) and methyl orange (MO) dyes demonstrated that the photocatalytic activity raise with a rise in the Ce-Ti molar ratio, as observed in 0.5 Ce-Ti/Cf. The composites’ effective photocatalytic activity with sunlight is owing to a synergistic effect between Cf and Ce-Ti NPs caused by a significant photogenerated carrier separation in the composites. Overall, the findings indicate that the addition of hydrogen peroxide could accelerate the organic molecule photodegradation with Ce-Ti when exposed to sunshine. Eventually, the conclusions of the current work participate in enhancing photodegradation purposes for the production of low-cost water in polluted provinces like (oxidation and industrial bonds), wherever water pollution is generally a serial with the rising problem.

## Data Availability

Research data can be obtained from the corresponding author through email.
